# Multi-scenario photoacoustic endoscopy for *in vivo* functional imaging

**DOI:** 10.1016/j.pacs.2025.100750

**Published:** 2025-07-05

**Authors:** Xiao Liang, Yuanlong Zhao, Linyang Li, Hongdian Sun, Wei Qin, Tingting Li, Heng Guo, Weizhi Qi, Lei Xi

**Affiliations:** a Department of Biomedical Engineering, Southern University of Science and Technology, Shenzhen, Guangdong 518055, China; b Guangdong Provincial Key Laboratory of Advanced Biomaterials, Southern University of Science and Technology, Shenzhen, Guangdong 518055, China; c Faculty of Health Sciences, University of Macau, Macau SAR 999078, China

**Keywords:** Endoscopy, Functional imaging, Photoacoustic microscopy, Ultra-fast scanning, Multi-view

## Abstract

Optical endoscopy has been extensively used in clinical screening and diagnosis of internal diseased organs. Photoacoustic endoscopy, one of the rapidest evolving optical endoscopies, combines rich optical contrasts with high spatial acoustic resolving capability at a considerable penetration depth. However, implementing high-speed, large field-of-view photoacoustic endoscopy in arbitrarily shaped biological tracts and cavities remains a challenge. Here, we develop a miniaturized, multi-view photoacoustic endoscope (Multi-PAE) that integrates a micro-optical scanner and a folded optical path within a capsule-sized probe. The probe features multiple interchangeable imaging interfaces in different orientations to image diverse tracts and cavities. We propose a compound double spiral resonant scanning (CDSRS) mechanism to enable the optical scanner to perform stable and uniform resonant scanning over a large field-of-view. We demonstrate the multi-scenario functional imaging applicability of Multi-PAE in rat rectums, rabbit cervices, and entire human oral cavities.

## 1 Introduction

Optical endoscopy has become an indispensable tool in clinical diagnosis and surgical interventions, providing direct visualization of internal tracts and cavities of hollow organs [1], [2]. Standard-of-care techniques such as white light endoscopy [3] (WLE) and narrow-band imaging [4], [5], [6] (NBI) enable the assessment of mucosal patterns and microvascular architectures on tissue surfaces, facilitating the detection of malignancies and guidance of therapeutic procedures. The advancement of miniaturized endoscopes [7], [8] offers high flexibility and superior resolving capabilities [9], [10]. These systems have further enhanced non-invasive monitoring of narrow organs and precise disease detections, reducing the risk of severe adverse events associated with surgical trauma and increasing the tolerance for a broader population [11], [12].

Among various optical modalities, photoacoustic endoscopy (PAE) offers unique features for visualizing structures and functions of microvasculatures [13] with a considerable penetration depth [14], [15]. PAE leverages the photoacoustic effect [16], [17], where laser pulses induce ultrasonic waves through thermoelastic expansion, to achieve high-resolution imaging of tissue structures [18], [19] and functions [20]. PAE is capable of effectively detecting abnormal blood oxygen level and angiogenesis-related vascular variations, which are critical biomarkers for early diagnosis of diseases [21], [22], [23]. Unfortunately, existing PAE systems still face challenges hindering further clinical translation. External mechanical-scanning-based PAE typically uses an electric motor to rotate the laser beam for acquisition of two-dimensional cross sections. To enable volumetric imaging, a linear translation stage is commonly used to execute a constant pullback translation of the imaging probe during sequential B-scan acquisition [24], [25], [26], [27]. This scanning strategy allows for the development of ultrasmall probes with a large angular field-of-view (FOV), which have been successfully applied to examining mouse thoracic aortas [28], rat rectums [26], [29], and rabbit esophagus [30]. Despite the excellent performance in the inspection of tubiform tracts and cavities in small animals, it is challenging to apply these probes in non-tubiform ones [23], [24]. External optical scanning strategies based on all-optical detection offer the highest axial resolution and superior three-dimensional images [31], [32]. Nevertheless, the minute-level frame rate and long rigid probe length make it challenging for *in situ* imaging of narrow or tortuous cavities. Compared with external scanning mechanisms, microelectromechanical system (MEMS)-based internal scanning mechanisms can acquire three-dimensional images within a single scanning sequence, enabling high-speed and low-distortion visualization of non-tubiform structures. To achieve internal optical scanning inside miniaturized probes, MEMS scanners are the key devices [23], [33], [34], [35]. However, due to the limited tilting angle of MEMS mirror, achieving a large FOV usually requires design compromise in probe length [36], which limits the maneuverability and applicable scenarios within the body. In addition, sampling inhomogeneity of high-speed MEMS scanning mechanisms leads to uneven heat distribution across the FOV and degraded image quality, posing risks of thermal damage and inaccurate diagnosis [35]. These limitations underscore a critical gap between existing PAEs and clinical needs of versatile endoscopic devices.

To address such gap, we present a multi-view photoacoustic endoscope (Multi-PAE) designed for multi-scenario functional imaging. Multi-PAE features a capsuled probe with three switchable interfaces for frontal view (0°), oblique view (30°), and lateral view (90°) imaging. This configuration enables adaptability to diverse tissue surfaces and anatomical scenarios. To achieve miniaturization, we develop a new package for the MEMS scanner that reduces the original packaged size by half. The optimized arrangement of miniaturized optical-acoustic components enables Multi-PAE to attain a diameter of 5.5 mm and a rigid length of 16 mm, facilitating navigation through narrow and complex anatomical pathways. To avoid sampling inhomogeneity and heat accumulation, we implement a compound double-spiral resonant scanning (CDSRS) strategy by integrating Archimedes and Fermat spirals. The CDSRS achieves a near-uniform scanning pattern and large FOV at the resonant frequency using the MEMS scanner. This approach delivers a FOV of 2 mm in diameter at a temporal resolution of 6 volumes per second. We validate the non-invasive diagnostic capabilities of Multi-PAE through *in vivo* functional imaging of rat rectums and rabbit cervices. Continuous assessment of oxygen saturation (sO_2_), total hemoglobin concentration (C_HbT_) and vascular morphological parameters during the progress of hemodynamic changes in rectal and cervical vasculatures were conducted to validate the *in vivo* imaging performance of the probe. In addition, combining with an ultrathin WLE and a flexible control handle, Multi-PAE enables real-time imaging of the entire oral cavity of volunteers, including lips, gingivae, tongues, cheeks, and oropharynges, to show the clinical feasibility.

## 2 Results

### 2.1 Design of the imaging system and probe

**Fig. 1:**
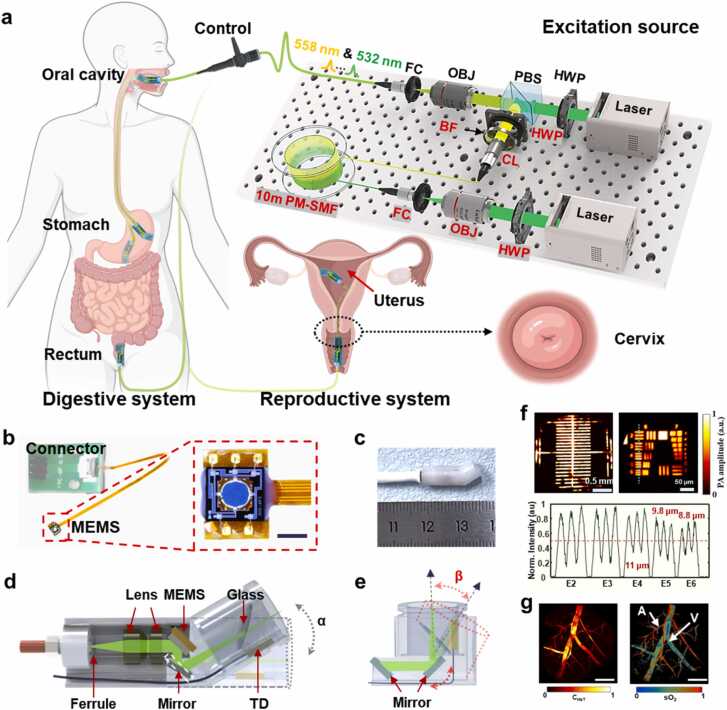
Multi-PAE system and probe design. a The conceived schematic of the Multi-PAE system. The PA excitation source employs two 532 nm lasers to generate a merged beam with 532 nm and 558 nm (see Methods for details). Multi-PAE is designed for imaging various organs of the digestive and reproductive systems. FC, fiber coupler; OBJ, objective; PBS, polarization beam splitter; HWP, half-wave plate; BF, bandpass filter; CL, collimator; PM-SMF, polarization-maintaining single-mode fiber. *In vivo* imaging was performed on the rat rectum, rabbit cervix, and human oral cavity to validate the system across multiple anatomical sites. b Photographs of the FPCB-packaged MEMS scanner and connector. Scale bar, 1 mm. c A photograph of a fully assembled 30°-view probe. d, e The 3D schematic of the probe and multi-view imaging interfaces (see Supplementary Fig. S2 for details). TD, transducer; α and β, interface adjustable angles. f Images of a reticle and a 1951 USAF target captured by the 30-degree oriented probe, and the profile across the elements 2–6 of group 6 indicated by the dashed line. (Supplementary Fig. S4 illustrates the comparative performance evaluation of the side-view and front-view probes.) g Representative images of hemoglobin concentration (C_HbT_) and oxygen saturation (sO_2_) of mouse intestinal blood vessels. A, artery; V, vein. Scale bar, 0.5 mm. a Partially created with BioRender.com released under a Creative Commons Attribution-NonCommercial-NoDerives 4.0 International license (https://creativecommons.org/licenses/by-nc-nd/4.0/deed.en).

Fig. 1a illustrates the conceptual schematic of the imaging system. The system consists of two key modules: the laser source module (see Methods for details) and the endoscopic probe module. The laser source module comprises two 532-nm nanosecond lasers to yield a 532-nm/558-nm dual-wavelength excitation. The 558-nm laser pulse is generated from a seed 532-nm laser *via* stimulated Raman scattering (SRS) effect. The dual-wavelength excitation source provides different absorption coefficients for oxygenated and deoxygenated hemoglobin, enabling the derivation of sO_2_ (Supplementary Note 1). As oxyhemoglobin and deoxyhemoglobin exhibit nearly identical absorption at 532 nm, we can determine the relative change of C_HbT_ from the photoacoustic (PA) amplitude at 532 nm [37]. For human oral imaging, the endoscopic probe module incorporates a Multi-PAE probe and a customized ultra-thin WLE in a control handle. Full details of the system are provided in the Methods section.

To reduce the size of the probe, we designed a flexible printed circuit board (FPCB) to optimize the MEMS packaging (Supplementary Fig. S1a). One end of the FPCB with the dimensions of 3.3 × 2.2 × 0.6 mm^3^ serves as the bonding base to offer rigid support for the MEMS chip. Another end of the FPCB is connected to an adapter to transmit driving signals. The MEMS chip is secured to the bonding base with UV glue and epoxy, and connected to the pins on the base with gold wires (Supplementary Fig. S1b**,**c). The overall size of the FPCB-packaged MEMS scanner is 3.3 × 2.2 × 1.25 mm^3^ (Fig. 1b). Based on the miniaturized MEMS scanner, we developed a capsule-sized probe featuring interchangeable imaging interfaces with various orientations (Fig. 1c, Supplementary Fig. S2, Supplementary Note 2). The shared components include a ceramic ferrule, two lenses and a MEMS scanner to enable laser focusing and 2D optical scanning (Fig. 1d). The interchangeable imaging interfaces are geometrically aligned and connected to the shared probe body, creating a folded optical path to maximize the scanning range within the compact probe. In front-view and 30°-view probes, a specific angled mirror on the interface directs the MEMS-scanned laser beam onto the target. In the side-view probe, two mirrors function in tandem to reflect the laser onto the target (Fig. 1e). By adjusting the mirror angles, we can re-orient the laser beam to accommodate interfaces of various views, enhancing the flexibility of the probe.

The Multi-PAE probe provides a circular FOV with a diameter of over 2 mm and a lateral resolution of 8.8 μm, which is sufficient for endoscopic vascular imaging (Fig. 1f). To further validate the performance of different interfaces, we affixed leaf veins to the inner surface of a pear-shaped mold and imaged them across different regions (Supplementary Fig. S3). Different interfaces showed superior imaging capability in different regions. Additionally, we imaged intestinal blood vessels in living mice and calculated corresponding sO_2_, confirming the functional imaging capability of Multi-PAE (Fig. 1g).

### 2.2 Compound double spiral resonant scanning strategy

**Fig. 2:**
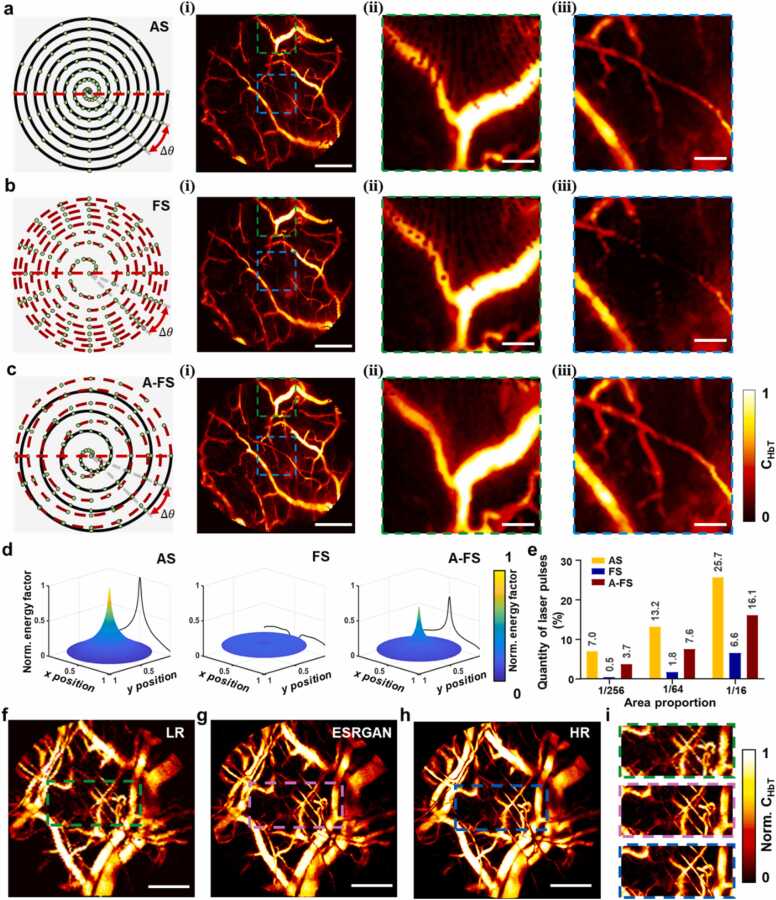
Compound double spiral resonant scanning and image restoration. a The schematic of Archimedes spiral (AS) scanning. The enlarged views taken from the edge (ⅱ) and the central region of the image (ⅲ) show dense sampling in the center and sparse sampling at the edge. b The schematic of Fermat spiral (FS) scanning. The enlarged views taken from the edge (ⅱ) and the central region of the image (ⅲ) show sparse sampling in both the center and edge regions. The vascular images (ⅰ) in (a) and (b) have 512 × 512 pixels. c The schematic of compound double spiral scanning (A-FS). A-FS combines AS and FS to form a uniform sampling pattern. The vascular image (ⅰ) has 512 × 512 pixels, similar to (ⅰ) in (a) and (b). The enlarged views taken from the edge (ⅱ) and the central region of the image (ⅲ) demonstrate significantly improved image quality with uniform sampling. Scale bars in (ⅰ), 0.5 mm; Scale bars in (ⅱ) and (ⅲ), 0.1 mm. d Comparison of energy distributions for different scanning mechanisms. The energy factor is defined as normalized log_10_(sampling points per pixel+1). The x-axis and y-axis represent the normalized position coordinates of the image and the z-axis is the normalized energy factor. The A-FS scanning optimizes energy distribution, thereby preventing heat damage in the central region. e Percentage of laser pulses (sampling points) within different diameters at the center of the scan area. Within the area of 1/256 in the center, AS scanning provides 7 % laser pulses, whereas A-FS reduces the number of pulses to 3.7 %. Within the area of 1/64 in the center, AS scanning provides 13.2 % laser pulses, while A-FS reduces the number of pulses to 7.6 %. f-h Sparsely-sampled, restored and fully-sampled vascular images at the same region. i Enlarged images of the dashed boxes in f, g, h for comparison. Scale bars, 0.5 mm.

We have demonstrated that double spiral resonant scanning (DSRS) can balance the temporal resolution, FOV, and fidelity in MEMS-based optical imaging systems [35]. However, it suffers from inhomogeneous sampling, where central regions are oversampled and marginal regions are sparsely-sampled. To address this issue, we proposed a CDSRS strategy, which integrated Archimedes spiral (AS) and Fermat spiral (FS) trajectories. Both AS and FS could enable undistorted resonant MEMS scanning by maintaining a constant angular velocity, acquiring an identical number of sampling points spaced at uniform angular intervals (Fig. 2a-c). Consequently, the AS trajectory yields dense internal sampling with sparsely-sampled edges, while the FS trajectory produces sparse internal sampling with densely-sampled edges. In addition, the driving waveforms for AS and FS could align smoothly, allowing for continuous, distortion-free compound Archimedes-Fermat spiral (A-FS) scanning (Supplementary Fig. S5). The A-FS scanning pattern interweaves AS and FS trajectories, increasing the number of effective sampling points and enhancing sampling uniformity across both central and edge regions, thereby yielding a better image quality (Supplementary Fig. S6). Comparative imaging of mouse intestinal vessels was conducted using AS, FS, and A-FS modes, with identical pixel counts. To demonstrate the raw imaging performance, no post-processing was applied to the images. Magnified views of the central and marginal regions reveal significant undersampling at the edges of AS scanning and in the central region of FS scanning (Fig. 2a(i)-b(iii)), while A-FS scanning effectively mitigates these issues (Fig. 2c(i)-c(iii)).

To quantify the differences among the three scanning trajectories, we analyzed the distributions of pixel-filled density along the diameter of the scanning domain (the red horizontal dashed lines in Fig. 2a-c). Compared to AS and FS scanning, A-FS scanning exhibits a more uniform spatial sampling rate, providing larger fully sampled areas and improved filling density at the edge (Supplementary Fig. 6h, Supplementary Fig. S7). Additionally, A-FS scanning reduces the number of sampling points in the central region, thereby minimizing energy accumulation in this area (Fig. 2d). A quantitative analysis of laser pulses within various diameters at the center of the scan region reveals that A-FS scanning decreases the number of laser pulses in the central area by approximately 50 % compared to AS scanning (Fig. 2e), effectively mitigating the risk of thermal damage, while still maintaining the image quality.

To enable the MEMS scanner to sweep in a resonant state and further improve the imaging speed, we decreased the number of sampling points during the scanning, resulting in inadequate sampling. We restored the image using a resolution-enhanced algorithm based on enhanced super-resolution generative adversarial networks (ESRGAN) (Supplementary Note 4, Supplementary Fig. S8) [38]. We trained the model using fully-sampled and corresponding sparsely-sampled PA images obtained by the A-FS scanning. Fig. 2f-h shows the sparsely-sampled, restored and fully-sampled images, respectively. The close-up views (Fig. 2i) show smoother vessel boundaries and fewer sparse sampling artifacts in the restored image, whereas the corresponding area in the sparsely-sampled image is blurred.

### 2.3 *In vivo* rectal imaging in rats

**Fig. 3:**
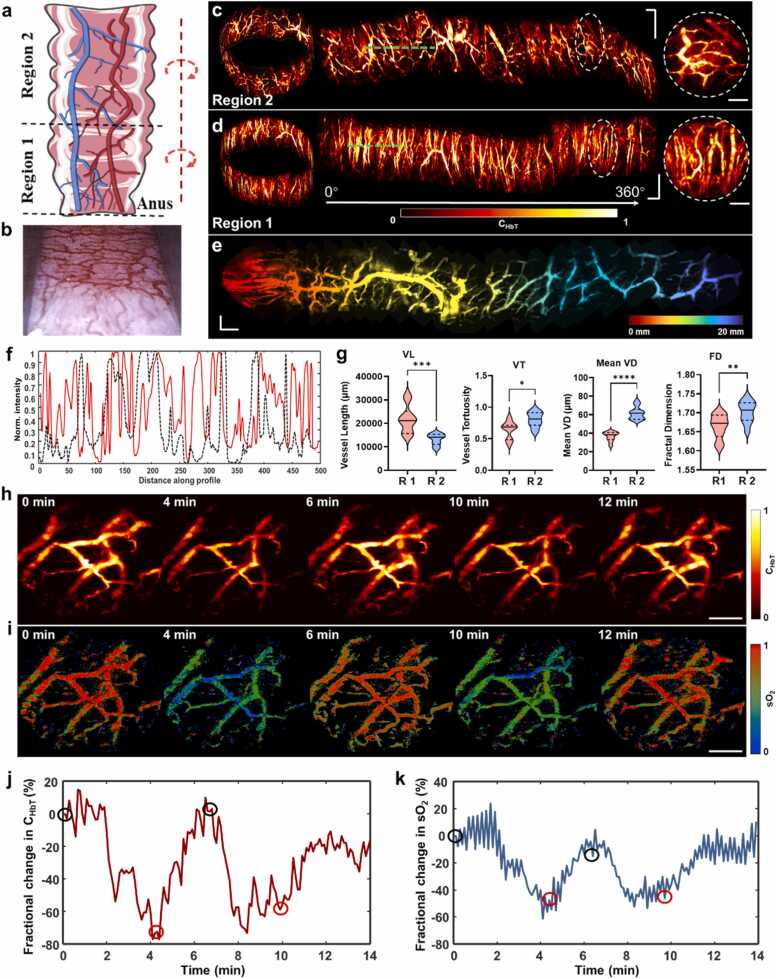
Endoscopic imaging of vascular anatomy and functions in rat rectums. a The schematic diagram of the rat rectal vascular anatomy. b A photograph of a typical rat rectum. c A 3D rendering and corresponding 360° angular FOV of the vasculature in region 2 of a rat rectum. The enlarged image shows typical reticular blood vessels in region 2. d A 3D rendering image and corresponding 360° angular FOV of the vascular system in region 1 of a rat rectum. As shown in the enlarged image, most vessels arrange parallel to the longitudinal direction. e A rectal image obtained by continuous longitudinal scanning, presenting continuous changes in vascular anatomical patterns. f The profiles along the green dashed lines in (c) and (d). The vessels near the anus (red curve) are significantly denser than those in deeper areas (black dashed curve). g Quantitative comparison of vessels in regions 1 and 2. Data analysis was performed on 10 selected single-FOV images from the corresponding regions. R 1, region 1; R 2, region 2; VL, total vessel length; VT, vessel tortuosity; VD, vessel diameter; FD, fractal dimension. h, i C_HbT_ and sO_2_ images captured at various time points during the hypoxia and resuscitation process. j, k Quantitative fractional changes in C_HbT_ and sO_2_ over the entire experimental process. The black circle represents the time when isoflurane is switched on and the red circle represents the time when isoflurane is switched off. Scale bars, 0.5 mm. *, p < 0.05; **, p < 0.01; ***, p < 0.001, ****, p < 0.0001. Scale bars: 0.5 mm.

To demonstrate the *in vivo* imaging capability of the side-view probe, we performed endoscopic imaging across multiple areas of a rat rectum. We employed 200 kHz laser pulses to achieve a 6 Hz frame rate within a 2 mm FOV, effectively avoiding image distortion caused by probe movement and intestinal peristalsis. The side-view probe enabled targeted vascular imaging within 1 cm (region 1) and 1–2 cm (region 2) from the anus (Fig. 3a), revealing distinct vascular patterns in each region (Fig. 3b). We manually rotated the probe to capture sequential images, and stitched the images together to generate comprehensive 360° views of the blood vessels in two regions of the rectal wall (Fig. 3c,d). Each 360° image took approximately 30 s. The imaging results reveal markedly distinct vascular characteristics. As depicted in the spliced and detailed images within the dashed boxes, region 1 presents dense and longitudinally parallel blood vessels, while region 2 displays a sparse, grid vascular pattern. To further illustrate longitudinal variations in rectal vascular structure, we conducted a continuous scan over a 2 cm range from the anal verge by manually advancing the probe longitudinally (Fig. 3e, Video 1). The stitched result clearly reveals the transformation in vascular morphology along the rectum. The profiles along the green dashed lines in regions 1 and 2 illustrate the distinct differences in vascular density (Fig. 3f). For a quantitative comparison, we randomly selected ten frames from each region and analyzed total blood vessel length (VL), vessel tortuosity (VT), mean vessel diameter (MVD), and fractal dimension (FD) (Supplementary Note 3). The VL in region 1 is significantly greater than that in region 2, while VT, MVD, and FD are lower in region 1 compared to those in region 2 (Fig. 3g). These results demonstrate the superior capabilities of the side-view probe for detailed intestinal vascular imaging.

To further validate the ability to monitor sO_2_ fluctuations in rat rectums, we performed continuous imaging with isoflurane cycling on and off to induce physiological changes (see Methods). The representative images of C_HbT_ and sO_2_ depict hemodynamic response during the experiments (Fig. 3h,i). Following 4 min of deep anesthesia, the C_HbT_ level markedly decreased across the FOV. Similarly, the sO_2_ dropped significantly below normal value due to the sustained hypoxia. Upon discontinuation of isoflurane, both C_HbT_ and sO_2_ gradually returned to the normal physiological level. Re-administration of isoflurane for another 4 min led to a subsequent decrease in C_HbT_ and sO_2_, as shown in the images labeled at the 10th min. Isoflurane was subsequently terminated, and the vessels were monitored for an additional 4 min. Until the 12th min, the C_HbT_ and sO_2_ significantly rebounded and stabilized. To quantify variations in C_HbT_ and sO_2_, we took the average value of the first five frames as the baseline and calculated the fractional changes with respect to the baseline across the FOV. On the fluctuation curves (Fig. 3j,k), black and red circles denote the initiation and cessation of high-concentration anesthetics, respectively. Notably, during the first cycle of anesthesia (marked by the first black circle), C_HbT_ and sO_2_ did not drop immediately but oscillated for approximately 2 min. Conversely, during the second round of deep anesthesia, the rat rapidly became hypoxic and sustained hypoxia until the cessation of isoflurane, in which C_HbT_ and sO_2_ immediately dropped and remained at low levels. At four minutes post isoflurane cessation, sO_2_ returned close to the baseline, while C_HbT_ recovered to approximately 80 % of the baseline. The side-view probe non-invasively visualized the variation in C_HbT_ and sO_2_ within the rectum, offering potential functional insights into physiological processes.

### 2.4 Long-term *in vivo* monitoring of rabbit cervicitis

Cervicitis, inflammation of the cervix, can significantly alter the cervical physiology, leading to changes in vascular structures and functions. In this study, we employed Multi-PAE with a front-view probe to monitor the progress of cervicitis *in vivo* over a period of two weeks. Rabbits with phenol-induced cervicitis (see Methods) were selected as the animal model. We inserted the imaging probe into the vagina and advanced it toward cervical regions. Manual manipulation of the probe enabled continuous acquisition of microvascular images in a large region, allowing for monitoring of morphological and functional changes throughout the course of inflammation and subsequent recovery.

**Fig. 4:**
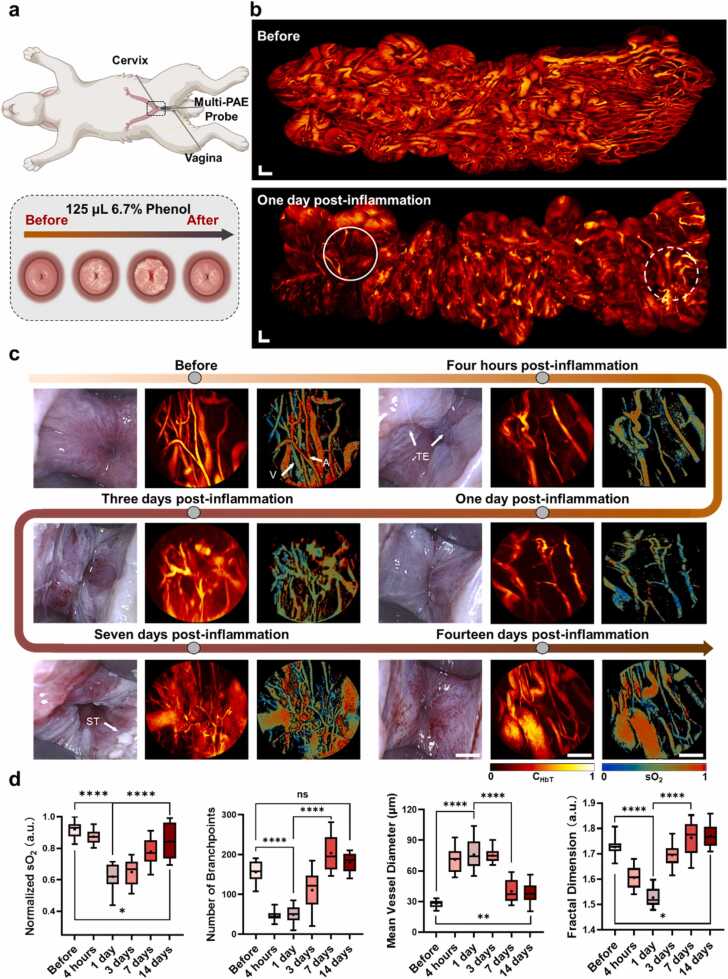
Long-term monitoring of Phenol-induced cervicitis in rabbits. a The schematic representation of the inflammation process in the rabbit cervix. The Multi-PAE probe was non-invasively inserted into the vagina until the front end contacted with the cervical inner membrane. b Stitched large-FOV images of the cervix before (top) and at one day post-inflammation (bottom). Changes in vascular structures indicate that acute inflammation cause alterations in vascular morphology (dashed circle) and a decrease in vascular density (solid circle). Scale bars, 0.5 mm. c PA and WLE images acquired at different time points. Images are arranged from left to right: WLE, PA, and sO_2_ map. Time points include before inflammation, four hours post-inflammation, one day post-inflammation, three days post-inflammation, seven days post-inflammation, and fourteen days post-inflammation. Scale bars for WLE images, 0.5 cm; for PA images and sO_2_ maps, 0.5 mm. d Quantification of vascular parameters shows changes in sO_2_, number of branch point, mean vessel diameter, and fractal dimension across different time points. Data analysis was performed on 12 randomly selected single-FOV images from the corresponding observation time points. These FOVs are consistent in size and do not overlap, ensuring reliable spatial representation. V: vein; A: artery; TE: tissue edema; ST: shedding tissues. Quantitative comparisons in (d) are represented by violin plots with quartile ranges (dotted) and medians (bold); ns, no significance, *, p < 0.05; **, p < 0.01; ****, p < 0.0001. a Partially created with BioRender.com released under a Creative Commons Attribution-NonCommercial-NoDerives 4.0 International license (https://creativecommons.org/licenses/by-nc-nd/4.0/deed.en).

Continuous monitoring of the vascular system in the inflammatory regions was conducted at six selected time points over two weeks. Fig. 4a illustrates the imaging procedure with Multi-PAE and provides a schematic representation of phenol-induced cervicitis. Video-rate imaging of the cervical region is shown in Video 2. Video 2 and Supplementary Fig. S9 illustrate that the vascular system within the cervix comprises multiple layers and exhibits random motions during the imaging process. The 3D imaging ability of the Multi-PAE is demonstrated in Supplementary Fig. S10, indicating vessel features among different layers. The CDSRS scanning strategy enables high-speed tracking of vascular dynamics and the stitching of images into a large FOV. By stitching these video frames, we obtain a large FOV image of the cervix, extending the area over 2 × 0.8 cm^2^ as shown in Fig. 4b and Supplementary Fig. S11. The stitching process is demonstrated in Video 3. This provides a more detailed and holistic view of the microvasculature at different time points (Fig. 4b, Supplementary Fig. S11, Video 4). At each time point, representative images captured by WLE and Multi-PAE are presented in Fig. 4**c**, monitoring both surface morphology and vascular architectures. To validate the dynamic monitoring capability of oxygen saturation, a hypoxia-recovery experiment was performed in a normal cervix where sO_2_ and C_HbT_ were captured as shown in Supplementary Fig. S12. As the isoflurane concentration increased, both sO_2_ and C_HbT_ levels decreased. Upon discontinuing isoflurane, these parameters gradually returned to baseline levels, indicating recovery to the normal state.

To comprehensively interpret the inflammatory process, we performed statistical analyses of the PA images to quantitatively record different vascular parameters (Fig. 4d). Before phenol injection, WLE images show a healthy cervix with a mucosal membrane that is smooth and moist. Multi-PAE provides detailed images of the vascular system with stable and clear vascular boundaries, and the functional map of sO_2_ indicates the normal cross-distribution of arterioles and venules (Fig. 4c). At four hours post phenol injection, tissue edema is observed on the mucosal surface (as indicated by the white arrows), marking the onset of acute inflammation [39]. This early stage is characterized by significant decreases in number of branch points (NBP) and FD, indicating vascular rarefaction due to the loss of microvascular structures (Fig. 4d). Despite the degradation of vascular density, the MVD increases, reflecting vasodilation and increased oxygen demand in the affected regions [40], [41]. A slight decrease in sO_2_ also suggests compromised oxygen delivery. At one day post-inflammation, a sharp decrease in sO_2_ is observed, while NBP and FD remain at low levels. Between three to seven days post inflammation, the vascular system undergoes angiogenesis, revealed by increased NBP and FD with decreased MVD. This reflects the formation of newborn, smaller blood vessels to restore oxygen supply and remove inflammatory mediators [42]. The increasing sO_2_ during this period suggests that these new blood vessels are contributing to tissue oxygenation, aiding in the recovery process [43], [44]. Through WLE images, we can observe an increase in shedding tissue, which is associated with excessive extracellular matrix deposition and the pruning of unnecessary vessels during tissue remodeling. From the final observation, the new vessels have stabilized with clear reconstructed boundaries. Tissue edema and the increased shedding of tissue disappear. 3D images of the cervical vasculature at different time points during cervicitis are shown in Supplementary Fig. S13, highlighting changes in the three-dimensional structure of the blood vessels caused by inflammation.

### 2.5 In vivo human oral imaging

**Fig. 5:**
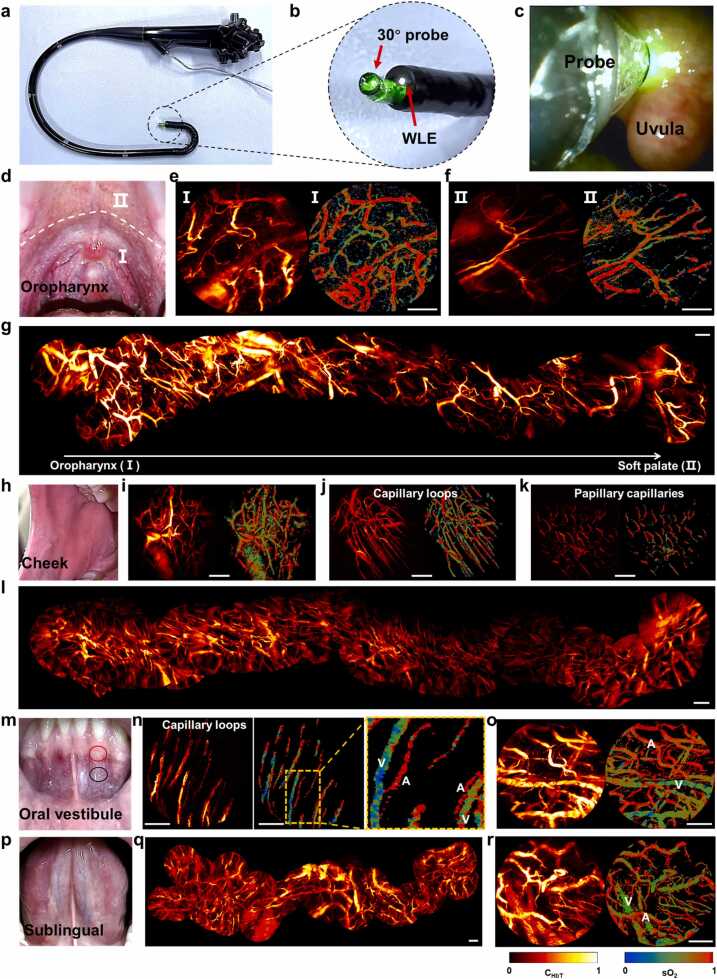
*In vivo* imaging of the human oral cavity. a A photograph of the customized control handle. A 30°-orientated probe and a WLE probe are mounted inside the handle. b The enlarged view of the integrated probe. c A photograph taken with the WLE during intraoral imaging. d A Photograph of the oropharynx of a volunteer. The white dashed line separates the oropharynx (Ⅰ) and soft palate (Ⅱ). e, f Representative hemoglobin concentration and oxygen saturation images in the oropharynx and soft palate. g Stitched roving scan from the oropharynx (Ⅰ) to the soft palate (Ⅱ), depicting continuous changes in vessel density. h A photograph of the cheek of a volunteer. i-k Typical vessel features and corresponding sO_2_ maps on the cheek. l Stitched roving data acquired by continuously scanning in the cheek (see Supplementary Fig. S15 for 3D vessel structures across varying layers). m A photograph of the oral vestibule area. The red circle indicates the gingival area, and the black circle indicates the mucosal area. n Linear capillary loops on the gums (red circle). The enlarged image clearly shows the venules and arterioles of unequal sizes. o Vascular structure and oxygen distribution on the mucosa near the gums (black circle). p A sublingual photo of a volunteer. q Stitched image acquired by continuous rapid scanning in the sublingual. r Representative sublingual hemoglobin and blood oxygen saturation maps. A, artery; V, vein. All scale bars, 0.5 mm.

The above demonstrations confirm the feasibility of the Multi-PAE probe for multi-scenario imaging in animals. To further evaluate the possibility of this approach for clinical imaging, we performed *in vivo* imaging of volunteer oral cavities. For oral imaging, we integrated a 30°-view Multi-PAE probe and a 1.2 mm diameter WLE into a customized control handle to guarantee the desirable maneuverability (Fig. 5a,b). The integrated imaging probe has a total diameter of approximately 12 mm, and can turn from −180° to 180 ° in two orthogonal directions. The WLE guided the probe to the target area and facilitated roving scans (Fig. 5c, Video 5). Additionally, we developed a cart-based acquisition system to further improve the flexibility in human oral imaging (Video 5). Label-free roving scans of the oropharynx, cheek, oral vestibule and sublingual areas were obtained by manipulating this probe to slowly move across these tissues.

The C_HbT_ and sO_2_ maps captured from the oropharynx (Ⅰ) exhibit notable different features compared to those from the soft palate (Ⅱ). The vessels in the oropharynx are significantly denser than those in the soft palate, which may be a manifestation of different functions (Fig. 5d-f). The roving scans from the oropharynx to the soft palate were stitched into a continuous image, revealing a transition from dense to sparse vessels (Fig. 5g). Vessels on the hard palate differ from those on the soft palate and pharynx, often appearing punctate vessels perpendicular to the tissue surface (Supplementary Fig. S14). We also imaged the vascular structures across multiple regions of the cheek (Fig. 5h). Vessels on the cheeks primarily appear in three forms: dense networks, elongated and papillary capillaries (Fig. 5i-k). Network-shaped and elongated vessels are usually interwoven with arterioles and venules, while papillary capillaries are predominantly arterial. The stitched image displays diverse vascular patterns on the cheeks (Fig. 5l, Video 6). A 3D image of the cheek area displays vascular features across layers, including papillary capillaries and transitions between tissue types (Supplementary Fig. S15, Video 7). Monitoring the oral vestibule holds considerable clinical potential, as it is often affected by ulcers, gingivitis, and various other disorders. Capillary loops in the gingiva facilitate arterial and venous oxygen exchange, showing a structured arteriovenous coexistence (Fig. 5m,n). Vessels in the mucosa near the gums are densely interlaced (Fig. 5o), resembling the vascular network inside the lips (Supplementary Fig. S16, Video 8). Microcirculatory alterations reflect infection severity and organ dysfunction. Sublingual vascular imaging allows non-invasive and direct observation of microcirculation, presenting C_HbT_ and sO_2_ in blood vessels (Fig. 5p-r, Video 8), potentially supporting the prompt assessment of microcirculatory changes across organ systems. Multi-PAE could feasibly detect various oral cavity disorders, from lips to oropharynx. Importantly, rapid imaging of targeted regions can be employed for early lesion screening, non-invasive monitoring, and follow-up examination.

## 3 Discussion and conclusion

In this study, we developed a capsule-sized, high-speed Multi-PAE capable of label-free functional microscopic imaging of biological tissues within diverse cavities. The Multi-PAE incorporated a miniaturized MEMS scanner with micro-optical and ultrasonic components in a multi-directional probe. Following thorough verification, we refined the probe dimensions to the optimal size (Supplementary Note 2), which are 5.5 mm in diameter and 16 mm in length. Decreasing the diameter necessitates a smaller MEMS scanner, which will compromise the aperture of incident laser beams, resulting in a lower resolution and a worse image quality. The length is mainly determined by the optical path. A considerable reduction in length will lead to a substantial reduction in scanning distance, hence compromising the imaging FOV. In the present study, the probe was oriented in three interchangeable views: frontal (0°), lateral (90°), and oblique (30°), whereas the interface can be customized to more orientations depending on the shape of targeted region to further enhance its *in vivo* clinical potentiality. In addition, the modular and replaceable interface design enables reuse of the scanning module, resulting in significant cost reduction. This cost-efficiency is beneficial for promoting the widespread adoption of the endoscope in both basic research and clinical practice.

The acquisition speed for non-invasive in-situ roving imaging was 6 Hz with the use of the CDSRS strategy. While conventional raster scanning produces evenly scanned images, it cannot achieve high-speed, high-fidelity resonant imaging due to abrupt mirror turning at the end of each B-scan. Previous double-spiral scanning enabled high-speed resonant scanning, however, it suffered from inhomogeneous sampling and potential heat accumulation [35]. The CDSRS strategy, integrating Archimedes spiral and Fermat spiral, allows high-speed resonant scanning with enhanced sampling homogeneity and uniform energy distribution. These features improved image quality and prevented potential photodamage. In addition, CDSRS extended the scanning angle over a limited scanning length within the compact probe, yielding a 2 mm circular FOV. These advancements hold substantial significance for clinical applications. The uniform distribution of laser energy enhances patient safety by mitigating the risk of tissue damage caused by localized high energy densities. Meanwhile, high-speed scanning reduces motion artifacts resulting from patient movement and enables real-time monitoring of physiological processes and treatment responses [45], [46], [47]. Notably, the imaging speed could be accelerated up to 12 Hz simply by employing 400 kHz lasers. Although this implementation will further reduce the motion artifacts during image acquisition, the increased laser energy may cause slight discomfort in the imaging area. Furthermore, it could cause nonlinear PA effects, influencing the precision of functional parameters analysis [48]. A potential solution is to employ high-sensitivity ultrasound detectors, such as optical sensors [49], [50], to reduce the minimum excitation energy.

The capability of flexible, high-speed, and label-free functional imaging, combined with high spatial resolution, offers substantial potential to expedite non-invasive disease diagnosis in arbitrary-shape organ cavities. The primary envisioned clinical application is the detection of disease within tubiform biological cavities, such as the early detection of rectal cancer and esophageal carcinoma. Cancer progression is often characterized by vascular proliferation and abnormal blood oxygen levels [51]. As demonstrated in this study, Multi-PAE enabled high-resolution rectal imaging, providing precise measurements of hemoglobin concentration and oxygen saturation fluctuations without motion artifacts, which are critical for early-stage lesion detection. Furthermore, the form factor of our miniaturized probe could allow *in situ* imaging in irregular biological cavities, which embodies the key advance. For example, it can perform microscopic investigations of the cervix and uterus for cervical carcinoma and endometrial cancer screening. We also demonstrated that Multi-PAE allows high-quality intraoral imaging, capturing both morphological and functional characteristics from the lips to the throat. This capability holds promise for identifying functional, physiological, and metabolic changes, introducing novel diagnostic features for stomatology. Given the crucial role of sublingual microcirculation monitoring in the physiological assessment of critically ill patients [52], Multi-PAE shows considerable promise for the utilization in intensive care settings. Another potential application is surgical guidance in open procedures, including brain, cardiac, intrauterine, and abdominal surgeries. It is noteworthy that, the miniaturized probe can be seamlessly integrated with commercial WLE, providing enhanced maneuverability for clinical use.

To enhance the performance of the Multi-PAE, we intend to integrate a micro-electrical motor or an electrically tunable lens to enable dynamic focal plane adjustment. This upgrade will allow the system to dynamically adapt to variations in vascular depth across heterogeneous tissue regions, thereby improving image consistency, optimizing signal acquisition, and expanding its applicability in complex clinical environments. Currently, the process of switching imaging interfaces incurs time overhead. Developing a probe capable of real-time adjustment of the interface orientation would significantly enhance its flexibility and adaptability across diverse clinical and research scenarios.

In summary, Multi-PAE introduces an ultra-compact, versatile imaging probe that enables rapid, high-fidelity, label-free functional imaging across a wide range of internal organ cavities. The advancements presented in this work address key limitations of current PAEs, particularly in terms of probe miniaturization, imaging speed, and adaptability, expanding the application scenarios. Our demonstrations suggest that Multi-PAE could serve as a valuable tool for no-invasion clinical examination, diagnosis and treatment assessment of diseases *via* accessible orifices such as the rectum, mouth and cervix, as well as for intensive care and surgical guidance.

## 4 Methods

### 4.1 MEMS bonding and probe fabrication

To miniaturize the scanner, we designed an FPCB for the electrothermal MEMS (OC-MI-T10, Wuxi WiO Technology Co., Ltd.) bonding. The packaged MEMS scanner mainly consists of an FPCB, a MEMS chip, and a glass sheet (Supplementary Fig. S1a,b). The packaging process involves four steps: Ⅰ. Fix the MEMS chip onto the FPCB using UV glue; Ⅱ. Connect MEMS chip pins to FPCB pins using gold wires; Ⅲ. Coat the gold wires with epoxy adhesive; Ⅳ. Seal the MEMS chip with a glass sheet (Supplementary Fig. S1c). Leveraging the miniaturized MEMS scanner, we calculated the theoretical minimum size of the endoscopic probe (see Supplementary Fig. S2a and Supplementary Note 2 for more details). Then, we designed a shared base and three interchangeable imaging interfaces. The fiber tip was secured in a ferrule on the shared base. A planoconvex lens (45–118, Edmund optics, USA) was used to collimate the laser beam emitted from the fiber tip. The collimated beam was focused by a planoconvex lens (45–273, Edmund optics, USA). The MEMS scanner reflected the focused laser beam and performed CDSRS scanning of the optical focus on the tissue surface. A customized mirror with dimensions of 2 × 2 × 0.5 mm^3^ was fixed on each interchangeable imaging interface. The mirror was fixed parallel to the MEMS mirror for the front-view interface and tilted at a 15° angle with respect to the MEMS mirror for the 30°-view interface. In the side-view interface, two mirrors reflected the laser beam sequentially to the imaging window. The interface was connected and adhered to the shared base with epoxy adhesive. By heating the epoxy adhesive, the existing interface could be removed and replaced with the desired one. A customized transducer with a central frequency of 10 MHz and dimensions of 2 × 2 × 0.5 mm^3^ was used to detect the PA signals. The glass sheet, placed at a 45° angle to the surface of the transducer, reflected the PA signals to the transducer.

### 4.2 Dual-wavelength pulsed laser source

We used two 532-nm pulsed lasers (VPFL- G- 20, Spectra-Physics) to achieve dual-wavelength PA excitation. The first laser provided 532-nm light, and another one pumped a Raman shifter to generate 558-nm light. In the Raman path, the polarization state of the laser was adjusted using a half-wave plate (GCL-060512, Daheng Optics) to maximize Raman shift efficiency in a 10-meter polarization-maintaining single-mode fiber (HB450-SC, Fibercore). The objective (PLN10X, Olympus) was mounted on a polarization-maintaining fiber launch (MBT621D/M, Thorlabs) to couple the pump laser into the fiber. The output laser was collimated using a fixed-focus collimation package (F220FC-532, Thorlabs) before being filtered by a bandpass filter (FBH560–10, Thorlabs). The 532-nm and 558-nm laser beams were combined by a polarizing beam splitter (GCCH-40202–532, Daheng Optics) and coupled to the fiber connected to the endoscopic probe. The laser pulse energy was approximately 150 nJ at both excitation wavelengths.

### 4.3 Animal preparation

All the experimental protocols were approved and supervised by the Animal Ethics Committee at the Southern University of Science and Technology (SUSTech) (SUSTech-JY202208007; SUSTech-JY202402001; SUSTech-JY202411125) and were conducted in accordance with the Laboratory Animal - General Requirements for Biosafety in Animal Experiments (GB/T 43051–2023). C57BL/6 mice (10–14 weeks old; weight, 20–25 g), Sprague-Dawley (SD) rats (12–20 weeks old; weight, 200–350 g), and New Zealand white rabbits (8–12 months old, weight, 2.5 kg-3.5 kg) were obtained from Guangdong Medical Laboratory Animal Center. All the animals were housed in their breeding rooms with a 12-hour light-dark cycle and proper food and water.

### 4.4 Mouse intestinal imaging

Six three-month-old female C57BL/6 mice were used for intestinal imaging experiments. Before experiments, the mice were fasted for 12 h. During imaging experiments, the mice were anesthetized with 1.5 % (v/v) isoflurane, and their body temperature was maintained at 37°C using a heating pad. The fur on the abdomen of mice was removed using depilatory cream. The abdominal skin was incised with disinfected surgical scissors to expose the intestines. Normal saline was instilled into the intestines of mice to maintain moisture and facilitate the transmission of PA signals. Then, the Multi-PAE probe was used to image intestinal blood vessels. After the experiment, the mice were euthanized.

### 4.5 Rat rectum imaging

Six four-month-old female SD rats were used for rectal imaging. The SD rats were fasted for 12 h, and an appropriate amount of medical glycerol was injected into the rectum of the rat to empty the intestine before rectal imaging. During the experiment, rats were fixed on a platform and a mixture of ultrasound coupling gel and deionized water was injected into their rectums to ensure ultrasound detection. The probe was wrapped in plastic wrap and then inserted into the rectum of the rat. A heating pad was placed under the rat to maintain its body temperature at 37°C.

Before the hypoxia experiment, rats were anesthetized with 2 % (v/v) isoflurane at a flow rate of 0.4 L/min. Once we started monitoring sO_2_, the isoflurane concentration was increased to 5 % (v/v) and the flow rate increased to 0.8 L/min to bring the rats into a state of hypoxia quickly. Following approximately 4 min of hypoxia, the isoflurane was turned off to allow the rats to breathe normally. After 2 min of resuscitation, the isoflurane was switched on again to re-induce hypoxia in rats. At the end of the 4-minute hypoxia experiment, isoflurane was turned off and rats were allowed to breathe freely until the experiments were terminated.

### 4.6 Rabbit cervicitis monitoring

Four New Zealand White rabbits (5–6 months old) were used for cervical imaging. The New Zealand White rabbits were anesthetized with 4 % isoflurane and placed in a supine position on the platform. A disinfected stainless-steel speculum was used to allow the probe to access the cervix easily. Physiological saline was used to keep the cervix moist and ensure the coupling of PA signals. We scanned the healthy cervix to acquire normal vascular images. To induce acute inflammation, we treated the rabbit cervix with 0.1 ml of saturated phenol solution (6.7 %) [53]. After 4 h, we used the endoscopic probe to image the modelling area. After the experiment, the rabbit was returned to the temporary breeding room for feeding. Rabbits were imaged again on days 1, 3, 7, and 14 following modelling. Before the hypoxia-recovery experiment, healthy rabbits were anesthetized with 3 % (v/v) isoflurane at a flow rate of 0.4 L/min. Once monitoring of sO_2_ began, the isoflurane concentration was increased to 5 % (v/v), and the flow rate was raised to 1 L/min to induce hypoxia rapidly. After approximately 4 min of hypoxia, the isoflurane was off, allowing the rabbits to breathe normally. At the end of the experiment, all the rabbits were euthanized using CO₂.

### 4.7 *In vivo* human oral imaging

Human participant procedures were reviewed and approved by Southern University of Science and Technology Institutional Review Board (20230033) and written informed consent was obtained from the participants. A Multi-PAE probe was specially assembled for human oral imaging and prohibited for animal experiments. A customized handle was employed to integrate the Multi-PAE probe and a customized WLE and control the movement of the probe in the oral cavity. Prior to the experiment, the probe was disinfected with 75 % medical alcohol and wrapped in sterile plastic wrap. Adult participants were asked to rinse their mouths with water before imaging experiments. The probe was moved around different areas in the oral cavity under the guidance of WLE.

### 4.8 Data processing and statistical analysis

The PA signals were first amplified by a pre-amplifier and then sampled by a data acquisition card (ATS9350, AlarzarTech) at a 250 MS/s sampling rate. All PA images were reconstructed using a customized script in MATLAB (R2022a, The MathWorks, Natick, USA). A vessel segmentation algorithm was used to analyze the hemodynamic changes (see Supplementary Note 3). VL is defined as the longitudinal dimension of vessel skeletons and the cumulative summation of lengths between the starting and ending points in each vessel segment. NBP signifies the termination of a vessel segment, where it bifurcates into two or more branches. NBP serves as an index of the vascular network's interconnectivity and the quantity of arborizations. For the points on the vasculature skeleton, the Euclidean distance between edge lines orthogonal to the centerline is defined as the vessel diameter (VD). MVD is calculated as the numerical average length of the normal line. The box-counting method is employed to approximate the FD by overlaying the binarized images with a grid of boxes with side lengths of r and denoting the number of nonempty boxes as N(r). FD is determined by the equation FD=log N(r)/ log (1/r). A measure of VT provides the ratio of the actual vascular path length (arc length) to the linear distance (chord length) between two branch points. The definition expression is formulated as VT=Arc Length / Chord Length. The statistical analysis was performed by GraphPad Prism (9.5.1, GraphPad Software, Boston, USA). Every parameter was calculated based on ten images and the numerical results were represented as mean value ± standard deviations. To determine statistical significance between the two experimental groups, an unpaired *t*-test or one-way analysis of variance (ANOVA) was performed with a significance level set at p < 0.05.

For multi-FOV stitching, adjacent FOVs were manually aligned by matching anatomical landmarks either within the overlapping regions or by applying rigid-body transformations (i.e., rotation and translation). Notably, the stratified architecture and peristaltic motion of mucosal blood vessels (Supplementary Figure S9), combined with changes in the distance between the imaging interface and the mucosal surface during movement (Supplementary Figure S17), can lead to morphological and structural variations of the same vessel across adjacent frames during continuous image acquisition. These variations may cause discontinuities in the vascular structures within the stitched images.

## CRediT authorship contribution statement

**Xiao Liang:** Writing – review & editing, Writing – original draft, Visualization, Validation, Software, Methodology, Investigation, Formal analysis, Data curation, Conceptualization. **Yuanlong Zhao:** Writing – original draft, Methodology, Investigation, Formal analysis, Data curation. **Linyang Li:** Software, Methodology. **Hongdian Sun:** Investigation. **Wei Qin:** Visualization. **Tingting Li:** Validation, Funding acquisition. **Heng Guo:** Project administration, Funding acquisition. **Weizhi Qi:** Project administration, Funding acquisition. **Lei Xi:** Writing – review & editing, Supervision, Resources, Project administration, Funding acquisition, Conceptualization.

## Code availability

All MATLAB scripts used to process and analyze data are available from the authors upon request. The deep-learning platform used in this study was adapted from a publicly available repository: https://github.com/xinntao/ESRGAN.

## Declaration of Competing Interest

The authors declare that they have no known competing financial interests or personal relationships that could have appeared to influence the work reported in this paper.

## Data Availability

Data will be made available on request.

## Footnotes

Appendix A Supplementary data associated with this article can be found in the online version at doi:10.1016/j.pacs.2025.100750.
