# Supplementary material for: Multi-scenario photoacoustic endoscopy for in vivo functional imaging
Source: Photoacoustics. 2025 Jul 5;45:100750. doi: 10.1016/j.pacs.2025.100750 (PMC12274738; doi:10.1016/j.pacs.2025.100750)
Supplement: Supplementary file 1 — Supplementary material [file mmc1.docx]

**Supplementary Information:**

**Multi-scenario photoacoustic endoscopy for *in vivo* functional imaging**

Xiao Liang,^1,3,^^†^ Yuanlong Zhao,^1,†^ Linyang Li^1^, Hongdian Sun^1^, Wei Qin^1^, Tingting Li^1^, Heng Guo^1^, Weizhi Qi^1^, Lei Xi^1,2^**^＊^**

^1^Department of Biomedical Engineering, Southern University of Science and Technology, Shenzhen, Guangdong 518055, China

^2^Guangdong Provincial Key Laboratory of Advanced Biomaterials, Southern University of Science and Technology, Shenzhen, Guangdong 518055, China

^3^Faculty of Health Sciences, University of Macau, Macau SAR 999078, China

† These authors contributed equally to this work.

*Corresponding author: xilei@sustech.edu.cn

**Supplementary Note 1:** Derivation of oxygen saturation.

**Supplementary Note 2:** The theoretical minimum diameter of the probe.

**Supplementary Note 3:** Quantitative analyses of vascular structures.

**Supplementary Note 4:** Deep learning algorithm for image restoration.

**Supplementary Figures**: S1-S17.

**Supplementary Video 1** High-speed longitudinal scan from the anus inwards rectum in a rat.

**Supplementary Video 2** Long-term monitoring of rabbit cervicitis with Multi-PAE.

**Supplementary Video 3** Demonstration of image stitching process.

**Supplementary Video 4** Comparison of vascular maps before and after cervicitis in rabbits.

**Supplementary Video 5** Roving imaging of human oropharynx using integrated Multi-PAE and white light endoscope.

**Supplementary Video 6** Continuous imaging of human cheek with integrated Multi-PAE and white light endoscope.

**Supplementary Video 7** 3D rendering of vascular structures in human cheek acquired by Multi-PAE.

**Supplementary Video 8** Roving imaging of human oral vestibule and sublingual blood vessels using Multi-PAE.

**Supplementary Note 1** **Derivation of oxygen saturation**

In this study, we calculate the oxygen saturation ($sO_{2}$​) and total hemoglobin concentration ($C_{HbT}$​) by analyzing photoacoustic intensities at two wavelengths of 532 nm and 558 nm (1, 2). $sO_{2}$​ is defined as the ratio of oxyhemoglobin concentration ($C_{HbO_{2}}$) to $C_{\mathrm{HbT}}$, which is the sum of $C_{\mathrm{Hb}O_{2}}$ and deoxyhemoglobin concentrations ($C_{\text{HbR}}$) (3):

$$\begin{aligned} \text{sO}_{2}=\frac{C_{\text{HbO}_{2}}}{C_{\text{HbO}_{2}}+C_{\text{HbR}}}\#\left( S1-1 \right) \end{aligned}$$

$C_{HbT}$ is defined as

$$\begin{aligned} C_{HbT}=C_{\text{HbO}_{2}}+C_{\text{HbR}}\#\left( S1-2 \right) \end{aligned}$$

Photoacoustic intensities at wavelengths of 532 nm and 558 nm are modeled as a combination of the molar extinction coefficients, optical fluence and concentration of hemoglobin at respective wavelengths as

$$\begin{aligned} S_{532}=\phi_{532}\cdot\epsilon_{532}^{\text{HbO}_{2}}\cdot C_{\text{HbO}_{2}}+\phi_{532}\cdot\epsilon_{532}^{\text{HbR}}\cdot C_{\text{HbR}}\#\left( S1-3 \right) \end{aligned}$$

$$\begin{aligned} S_{558}=\phi_{558}\cdot\epsilon_{558}^{\text{HbO}_{2}}\cdot C_{\text{HbO}_{2}}+\phi_{558}\cdot\epsilon_{558}^{\text{HbR}}\cdot C_{\text{HbR}}\#\left( S1-4 \right) \end{aligned}$$

where $S_{532}$ and $S_{558}$ denote the photoacoustic intensities at wavelengths of 532 nm and 558 nm. $\epsilon^{\text{HbO}_{2}}$*​* and $\epsilon^{\text{HbR}}$ denote the absorption coefficients of oxyhemoglobin and deoxyhemoglobin. $C_{\text{HbO}_{2}}$ and $C_{\text{HbR}}$ represent the concentrations of oxyhemoglobin and deoxyhemoglobin. $\phi_{532}$ and $\phi_{558}$ denote the local optical fluences. Substituting $S_{532}$ and $S_{558}$ into Eq. (S1-1), $\text{sO}_{2}$ can be calculated as

$$\begin{aligned} \text{sO}_{2}=\frac{\lambda\cdot\epsilon_{558}^{\text{HbR}}-\epsilon_{532}^{\text{HbR}}}{\lambda\left( \epsilon_{558}^{\text{HbR}}-\epsilon_{558}^{\text{HbO}_{2}} \right)+\epsilon_{532}^{\text{HbO}_{2}}-\epsilon_{532}^{\text{HbR}}}\#\left( S1-5 \right) \end{aligned}$$

where $\lambda=D_{532}/D_{558}$, with $D_{532}=S_{532}/\phi_{532}$ and $D_{558}=S_{558}/\phi_{558}$. We substitute the known molar extinction coefficients $\epsilon_{558}^{\text{HbR}}$ = 54164, $\epsilon_{558}^{\text{HbO}_{2}}$ = 33456, $\epsilon_{532}^{\text{HbR}}$ = 40584, and $\epsilon_{532}^{\text{HbO}_{2}}$ = 43876 (cm^-1^·M^-1^) into Eqs. (S1-5) (4), allowing for the calculation of $\text{sO}_{2}$. The derived parametersare then mapped to the vessel structures for further analysis.

**Supplementary Note 2 The theoretical minimum diameter of the probe.**

The diameter of the probe is primarily constrained by the dimensions of the MEMS scanner, as well as the sizes of lenses and mirrors. To determine the theoretical minimum diameter, we analyzed the layout of key components: the MEMS scanner (3.3 × 2.2 × 1.25 mm³), the focusing lens (3 mm in diameter and 1.08 mm in thickness), and the mirror (2 × 2 × 0.5 mm³). To achieve the smallest probe diameter, the distance between the MEMS scanner and the mirror must be minimized while maintaining an unobstructed optical path. As the laser beam is particularly susceptible to MEMS obstruction during scanning in the 30°-oriented probe, we selected this probe for the calculation. **Supplementary Note 2, Figure 1** presents a geometric sketch of the 30°-oriented probe, illustrating the critical relationships between the components and their arrangement. The following equations describe the probe configuration:

$$\begin{aligned} d_{1}=T_{1}\sin45^{\circ}+L_{1}\sin45^{\circ}\#\left( S2-1 \right) \end{aligned}$$

$$\begin{aligned} co=\frac{L_{2}}{2}\sin30^{\circ}+\frac{T_{2}}{2}\cos30^{\circ}\#\left( S2-2 \right) \end{aligned}$$

$$\begin{aligned} bc=d_{2}-co+\frac{L_{1}}{2}\sin45^{\circ}\#\left( S2-3 \right) \end{aligned}$$

$$\begin{aligned} \frac{bc}{\sin\theta_{1}}=\frac{{bc}^{'}}{\sin\theta_{2}}\#\left( S2-4 \right) \end{aligned}$$

$$\begin{aligned} \frac{{bc}^{'}}{\sin\theta_{3}}=\frac{bk}{\sin\theta_{4}}\#\left( S2-5 \right) \end{aligned}$$

$$\begin{aligned} d=d_{1}+d_{2}\#\left( S2-6 \right) \end{aligned}$$

Where *d_1_* is the width of the MEMS projection on the probe cross-section, *d_2_* is the distance between the edge of MEMS and the edge of the mirror, *T_1_* is the thickness of the MEMS, *L_1_* is the width of the MEMS, *L_2_* is the width of the mirror, *T_2_* is the thickness of the mirror. From these calculations, the theoretical minimum diameter d is approximately 3.6 mm.

**
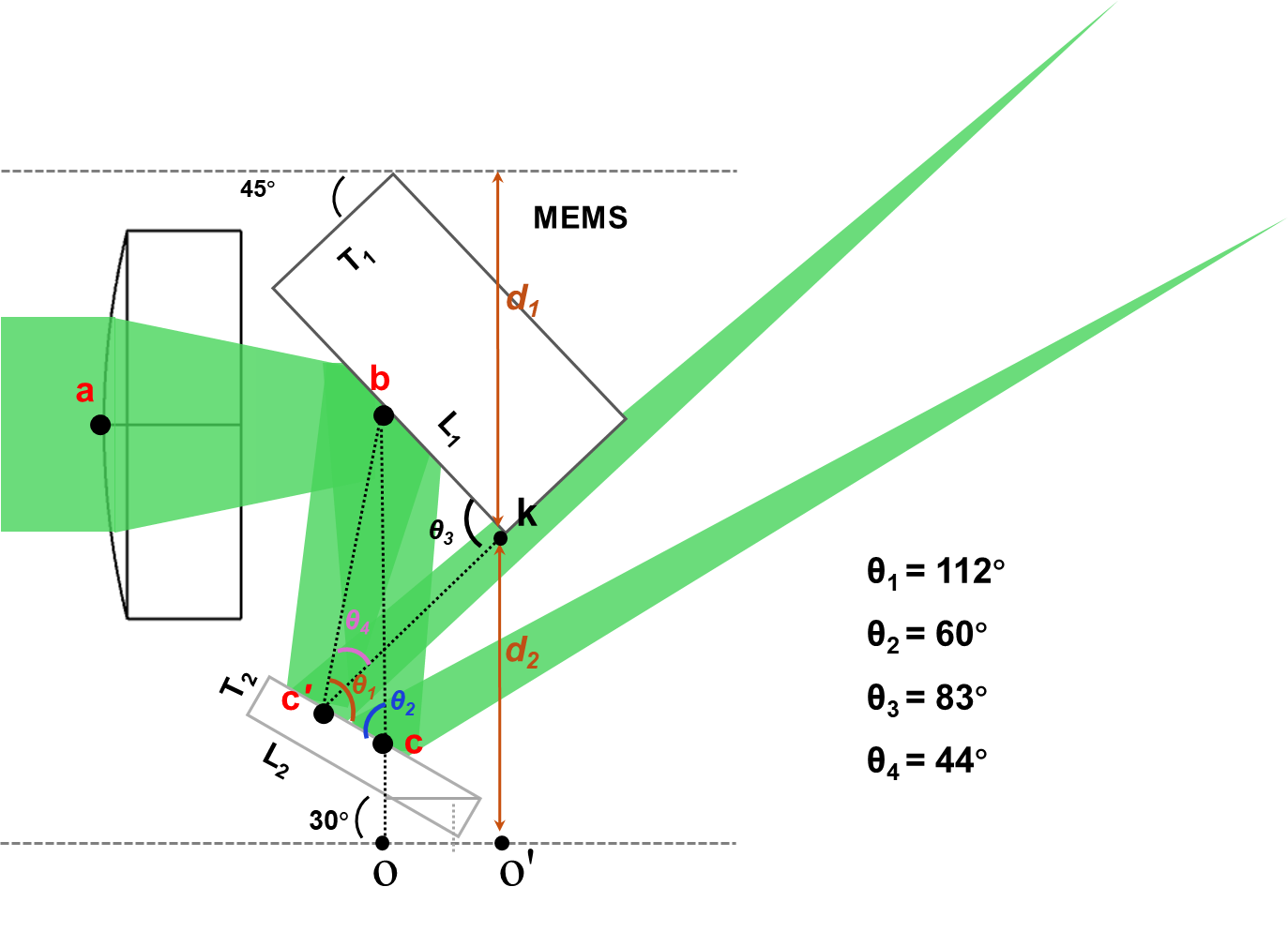
**

**Supplementary Note 2 Figure 1 | The geometric sketch of the 30°-oriented probe.** The packaged MEMS scanner has dimensions of 3.3 × 2.2 × 1.25 mm^3^, with a maximum MEMS mirror deflection angle of 4°. The mirror dimensions are 2 × 2 × 0.5 mm^3^.

**Supplementary Note 2 Figure 2** illustrates the cross-sectional view of the probe in a Cartesian coordinate system. Point A, defined as the midpoint of the MEMS edge, is set as the origin of the coordinate system. The distance between points A and B is 3.6 mm. The coordinates of points B, C, and D are (3.6, 0), (3.6, 1), and (0, 1.65), respectively.
The midpoint of the line segment CD, denoted as point E, is used to locate the perpendicular bisector of line segment CD. The perpendicular bisector intersects the x-axis at point O, which represents the center of the probe cross-section. Using principles of analytic geometry, the line passing through points O and E is described by:
$\begin{aligned} 5.54x-y-8.64=0 \#\left( S2-7 \right) \end{aligned}$

By solving it for the coordinates of point O, we determine O = (1.56, 0). The probe radius, defined as the distance from point O to point D, is approximately 2.27 mm. Considering a wall thickness of 0.5 mm, the minimum probe diameter is calculated to be approximately 5.5 mm. The length of the probe is largely determined by the focal lengths of the collimating and focusing lenses. Further reduction in probe length would adversely affect imaging resolution and range.

**
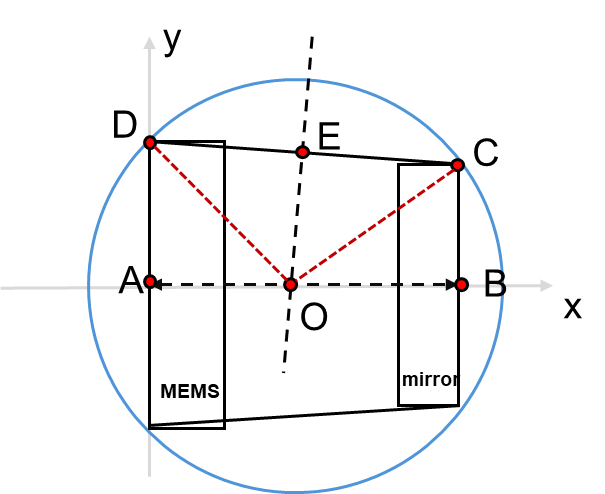
**

**Supplementary Note 2 Figure 2 | The cross-sectional schematic of the probe.** The centers of the MEMS scanner and the mirror are aligned along the same horizontal axis. The blue circle represents the outer boundary of the isosceles trapezoid formed by the MEMS scanner and the mirror.

**Supplementary Note 3 Quantitative analyses of vascular structures**

The segmentation of vascular regions begins by constructing a multi-scale representation of the raw intensity distribution. For each scale, defined by a parameter $\sigma$, the image $I(x,y)$ is firstly convolved with the Gaussian second-derivative kernel to produce a Hessian matrix $H_{\sigma}\left( x,y \right)$ (5). The Hessian at scale $\sigma$ is represented as

$$\begin{aligned} H_{\sigma}\left( x,y \right)=\left( \begin{matrix} \frac{\partial^{2}G_{\sigma}\left( x,y \right)}{\partial x^{2}} & \frac{\partial^{2}G_{\sigma}\left( x,y \right)}{\partial x\partial y} \\ \frac{\partial^{2}G_{\sigma}\left( x,y \right)}{\partial y\partial x} & \frac{\partial^{2}G_{\sigma}\left( x,y \right)}{\partial y^{2}} \end{matrix} \right)\#\left( S3-1 \right) \end{aligned}$$

where $G_{\sigma}\left( x,y \right)=I\left( x,y \right)*g_{\sigma}\left( x,y \right)$ and $g_{\sigma}\left( x,y \right)$ is a Gaussian kernel of standard deviation $\sigma$. By analyzing the eigenvalues $\lambda_{1}$ and $\lambda_{2}$ of $H_{\sigma}\left( x,y \right)$, vascular feature margins will be detected with $\left| \lambda_{1} \right|\leq\left| \lambda_{2} \right|$. To quantify the filtering process, we defined the following parameters as

$$\begin{aligned} R=\frac{\left| \lambda_{1} \right|}{\left| \lambda_{2} \right|},\quad S=\sqrt{\lambda_{1}^{2}+\lambda_{2}^{2}}\#\left( S3-2 \right) \end{aligned}$$

where *R* is the ratio and *S* is the Euclidean norm between eigenvalues. The vesselness measure at scale $\sigma$ is expressed as (6)

$$\begin{aligned} V_{\sigma}\left( x,y \right)=\left( 1-e^{-\frac{R^{2}}{2\alpha^{2}}} \right)e^{-\frac{S^{2}}{2\beta^{2}}}\#\left( S3-3 \right) \end{aligned}$$

where $\alpha$ and $\beta$ are parameters controlling the sensitivity to elongated structures. Evaluating $V_{\sigma}\left( x,y \right)$ over multiple scales $\sigma\in\{\sigma_{min},\ldots,\sigma_{max}\}$ and selecting the maximum response at each pixel location yield a final vesselness map:

$$\begin{aligned} V\left( x,y \right)=\max_{\sigma} V_{\sigma}\left( x,y \right)\#\left( S3-4 \right) \end{aligned}$$

Applying a threshold $\tau$ to $V\left( x,y \right)$ produces a binary mask $B\left( x,y \right)$, as shown in **Supplementary Note 3 Figure 1**. In the analysis process, the vascular area fraction ($\mathrm{VAF}$) is computed by taking the ratio of vessel pixels to the total image area A (7). If the image has dimensions $M\times N$, the VAF is given by:

$$\begin{aligned} VAF=\frac{\sum_{x=1}^{M} \sum_{y=1}^{N} B\left( x,y \right)}{A}\#\left( S3-5 \right) \end{aligned}$$

A skeletonization process reduces the binary vessel map to a one-pixel-wide skeleton $S(x,y)$. **Supplementary Note 3 Figure 1** indicates that branch points are identified as pixels in the skeleton where three or more vessel segments converge. The skeleton $S(x,y)$ is then segmented into individual segments by isolating sections between branch points in **Supplementary Note 3 Figure 1**, allowing for analysis of diameter and tortuosity. For a given segment, let $L_{1}$ be the actual path length (sum of pixel-to-pixel distances along the skeleton) and $L_{2}$ be the straight-line distance between its endpoints. The vessel tortuosity ($VT$) for that segment is defined as:$\begin{aligned} VTI=\frac{L_{1}}{L_{2}}\#\left( S3-6 \right) \end{aligned}$

**Supplementary Note 3 Figure 1** indicates the tortuosity map of $S(x,y)$. A distance transform is performed on the binary vessel image $B(x,y)$, assigning to each vessel pixel the shortest distance $D(x,y)$to the nearest background pixel. The mean vessel diameter at skeleton pixels can be calculated as

$$\begin{aligned} MVD=\frac{\sum_{x=1}^{M} \sum_{y=1}^{N} \text{D}\left( x,y \right)\cdot S\left( x,y \right)}{\sum_{x=1}^{M} \sum_{y=1}^{N} S\left( x,y \right)}\#\left( S3-7 \right) \end{aligned}$$

A box-counting method is employed to quantify the fractal dimension of the vascular network. The binary vessel image is covered with grids of varying box sizes $r$, shown in **Supplementary Note 3 Figure 1**. For each box size, $N(r)$ denotes the number of boxes that contain at least one vessel pixel. The fractal dimension $FD$ ​is estimated by analyzing the scaling relationship between $N(r)$ and $r$:

$$\begin{aligned} FD=-\frac{\Delta\log\left( N\left( r \right) \right)}{\Delta\log\left( r \right)}\#\left( S3-8 \right) \end{aligned}$$

By plotting $log(N(r))$ against $log(r)$ and calculating the slope of the linear fit, the fractal dimension $FD$​ is determined, capturing the intricate structural complexity of the vascular network.


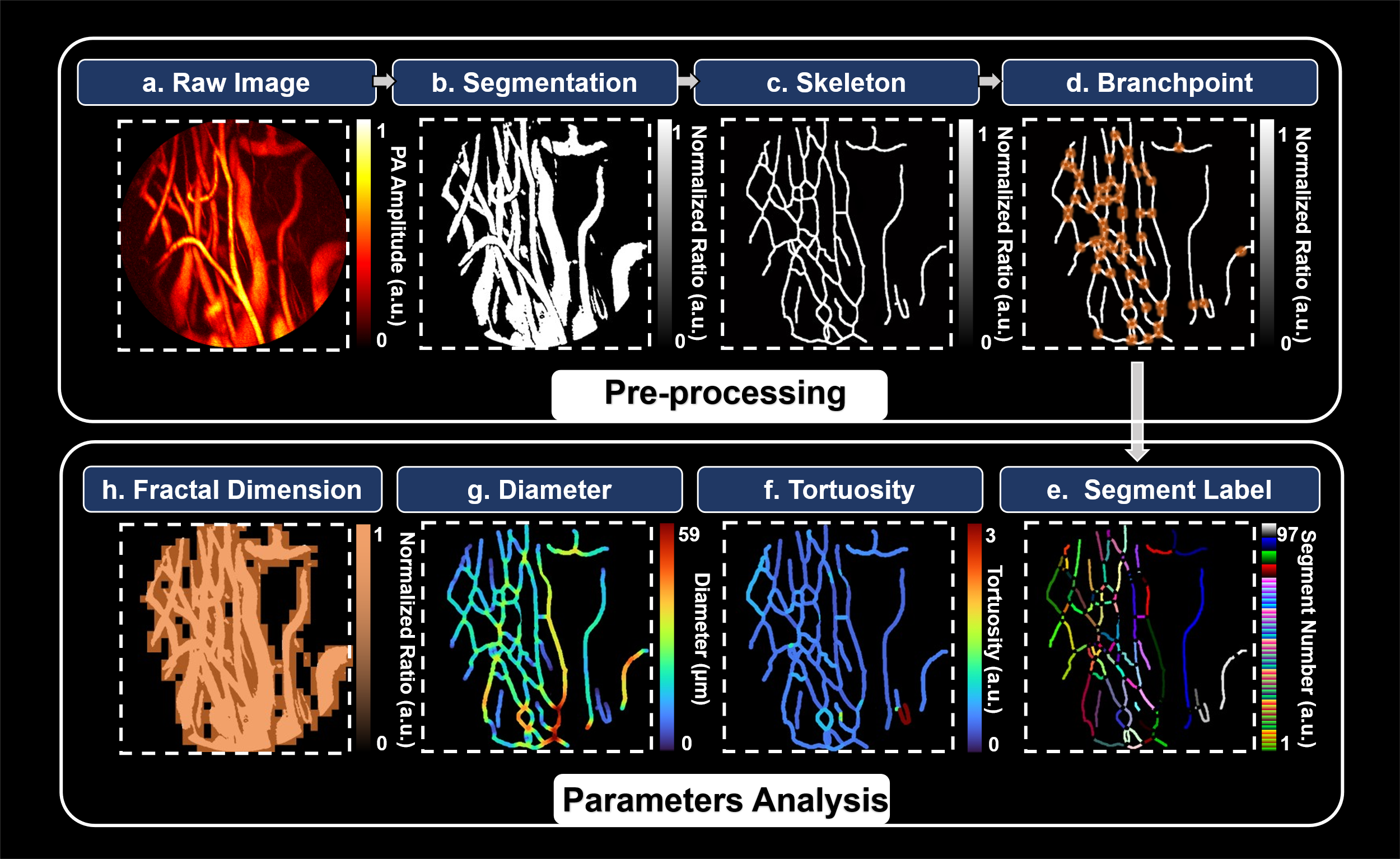


**Supplementary Note 3 Figure 1.** The flow chart of quantification of vascular morphological parameters. Pre-processing of the PA image: **a** A raw photoacoustic image of the vascular structure. **b** Binary segmentation of the vascular network. **c** Skeletonization of the segmented image to extract centerlines. **d** Identification of branch points within the vascular network. Parameter analysis: **e** Segment labeling for individual vessel segments with different colors. **f** Calculation of vessel tortuosity. **g** Vessel diameter measurement. **h** Box-counting fractal dimension analysis for network complexity evaluation, with a mask indicating vessel regions.

**Supplementary Note 4 Deep learning algorithm for image restoration.**

**4.1 Network Architecture**

In this work, we adopted a resolution enhanced algorithm based on enhanced super-resolution generative adversarial networks (ESRGAN) (8). This framework simultaneously employs two sub-networks: a generative network learns how to enhance the resolution for $512\times512$ images and a discriminative network returns an adversarial loss between the enhanced image and the corresponding ground truth. In the generator design (**Supplementary Fig. S7a**), we retained the high-level architecture design of ERGAN, and used 16 Residual-in-Residual Dense Block (RRDB), which combines multi-level residual networks and dense connections. Furthermore, we removed all batch normalization (BN) layers to improve generalization ability and reduce computational complexity and memory usage. In the discriminator design (**Supplementary Fig. S7b**), we used a VGG network (9), which contains 10 convolutional layers with an increasing number of 3×3 filter kernels, increasing by a factor of 2 from 32 to 512 kernels. The resulting 512 feature maps are followed by two dense layers and a final sigmoid activation function to obtain a probability for sample classification. Based on the relativistic GAN (10) and perceptual loss, we designed the loss function of the model. Specifically, the total loss for the generator is defined as:

$$\begin{aligned} L_{G}=L_{percep}+\lambda L_{G}^{Ra}+\eta L_{1}\#\left( S4-1 \right) \end{aligned}$$

where $L_{1}=\mathbb{E}_{x_{i}}\left\| G\left( x_{i} \right)-y \right\|_{1}$ is the content loss that evaluate the 1-norm distance between the recovered image $G\left( x_{i} \right)$ and the ground-truth $y$, the $L_{percep}$ is the perceptual loss, $L_{G}^{Ra}$ is the adversarial loss for generator based on relativistic GAN, and $\lambda$, $\eta$ are the coefficients to balance different loss terms.

The loss for the discriminator is given by:

$$\begin{aligned} L_{D}=L_{D}^{Ra}\#\left( S4-2 \right) \end{aligned}$$

where $L_{D}^{Ra}$ is the adversarial loss for the discriminators based on relativistic GAN.

**4.2 Training Details**

Following ESRGAN, all experiments were performed with a scaling factor of ×4 between low-resolution (LR) and high-resolution (HR) images. The generator is trained using the loss function described in Eq.(S4-1) with $\lambda=0.005$ and $\eta=0.01$. The learning rate is set to ${10}^{-4}$ and decayed by a factor of 2 every ${5\times10}^{4}$ iterations. Perceptual loss was calculated using the $L_{1}$ loss. We employed Adam optimizer (11) and alternately updated the generator and discriminator network until the model converges.

For training datasets, we used HR images acquired at 512 × 512 pixels, and corresponding LR images acquired at 128 × 128 pixels. The images were reconstructed uniformly as patches, and any unsampled pixels in the LR images were filled with zero values. We augmented the training dataset with random horizontal flips and 90-degree rotations. The model was trained using 1000 pairs of HR and LR images and tested using 100 pairs of HR and LR images.

As demonstrated in **Supplementary Figs. S7 c,d**, our results show that the deep learning algorithm can recover fine vessel details more effectively from a reduced number of pixels in the original images compared to the traditional interpolation algorithm. This suggests that images can be acquired at much lower scanning densities, providing a significant boost to the imaging frame rates.

**Supplementary Figures**

**
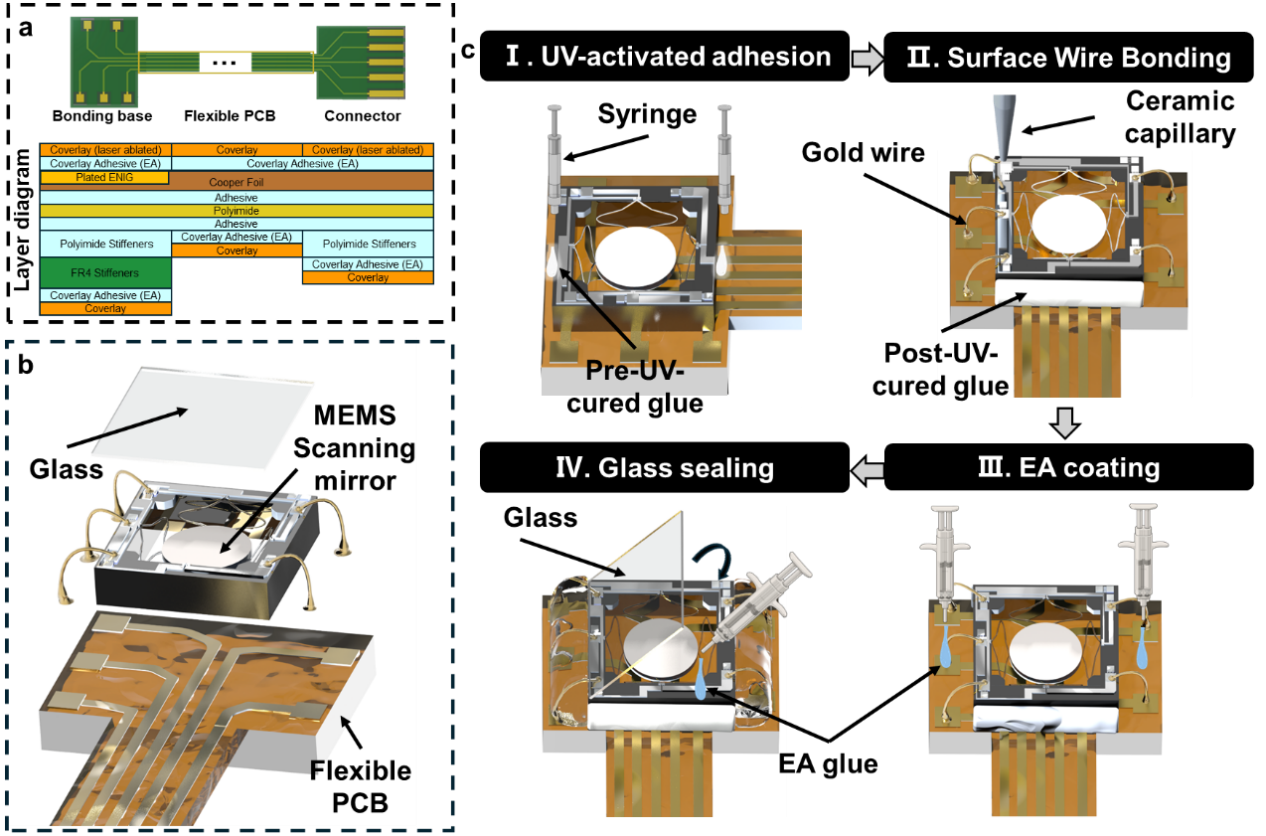
**

**Figure S1. Packaging process of the MEMS chip. a** The schematic representation of the flexible PCB, highlights the distinctions among different layers. PCB printed circuit board, UV ultraviolet, EA epoxy adhesive, FR flame retardant, number ‘4’ indicates woven glass-reinforced epoxy resin, ENIG electroless nickel immersion gold. **b** The overall structure of the miniature MEMS scanner. **c** The packaging workflow of the MEMS scanner. The MEMS chip was affixed on the bonding base of the flexible PCB using UV-cured glue. Then the bonding base and MEMS chip were interconnected *via* gold wires. To enhance the reliability, the MEMS scanner was further reinforced with epoxy adhesive and sealed with a glass sheet.

**
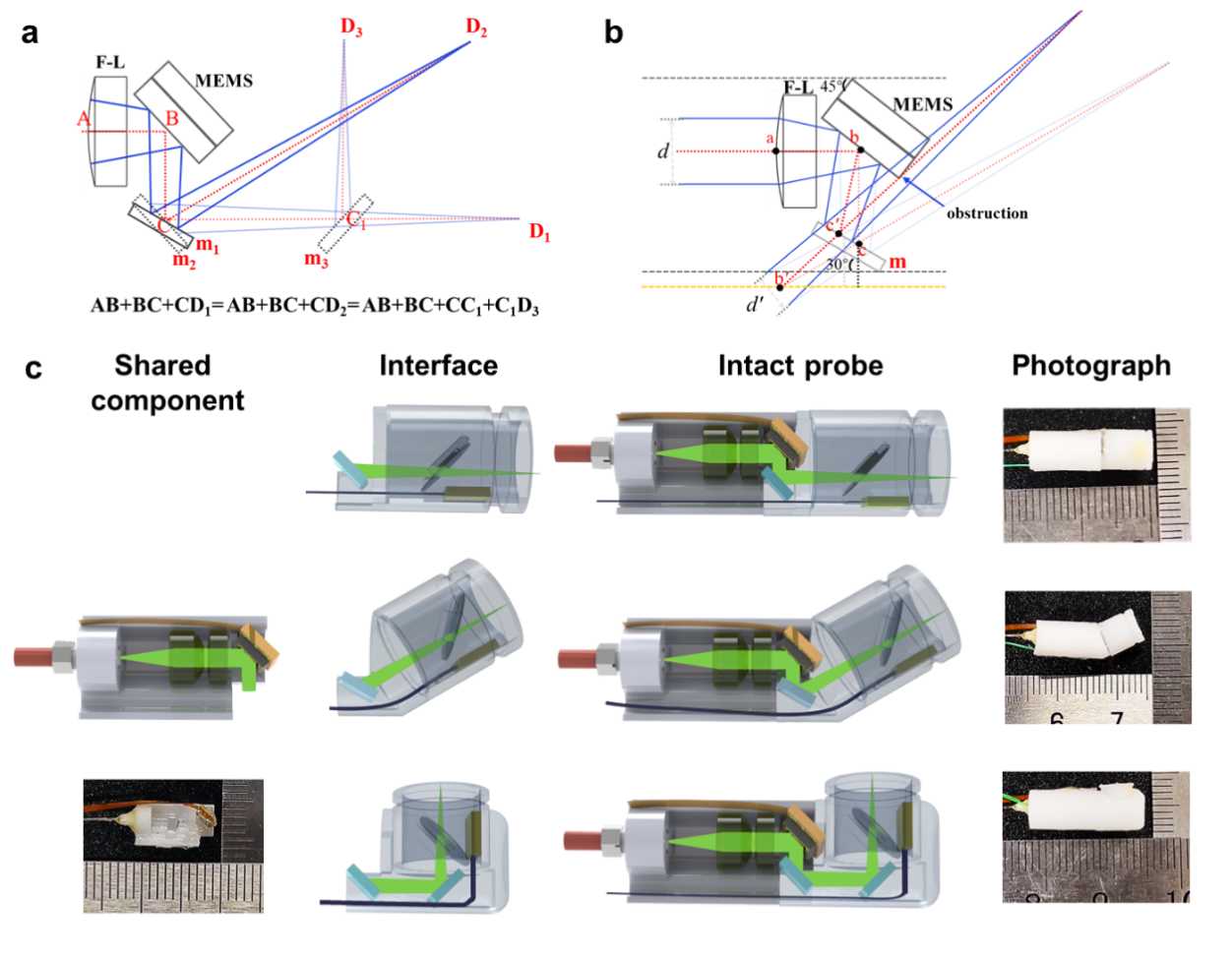
**

**Figure S2. Design of multi-view probes. a** The schematic illustration of the transition from the front-view to the side-view probes. The working distance of the focusing lens for different probes remains constant despite changes in configuration (AB+BC+CD_1_=AB+BC+CD_2_=AB+BC+CC_1_+C_1_D_3_). **b** Analysis of the optimal dimensions for the 30°-oriented probe (see **Supplementary Note 2** for details). **c** Configurations of the front-view (D1), 30°-view (D2), side-view (D3) probes and corresponding photos. Three differently oriented interfaces can be adhered to the shared component separately to form an intact probe. F-L, focus lens; m_1_, m_2_, m_3_:mirrors with tilting angles of 30°, +45°, and -45°.

**
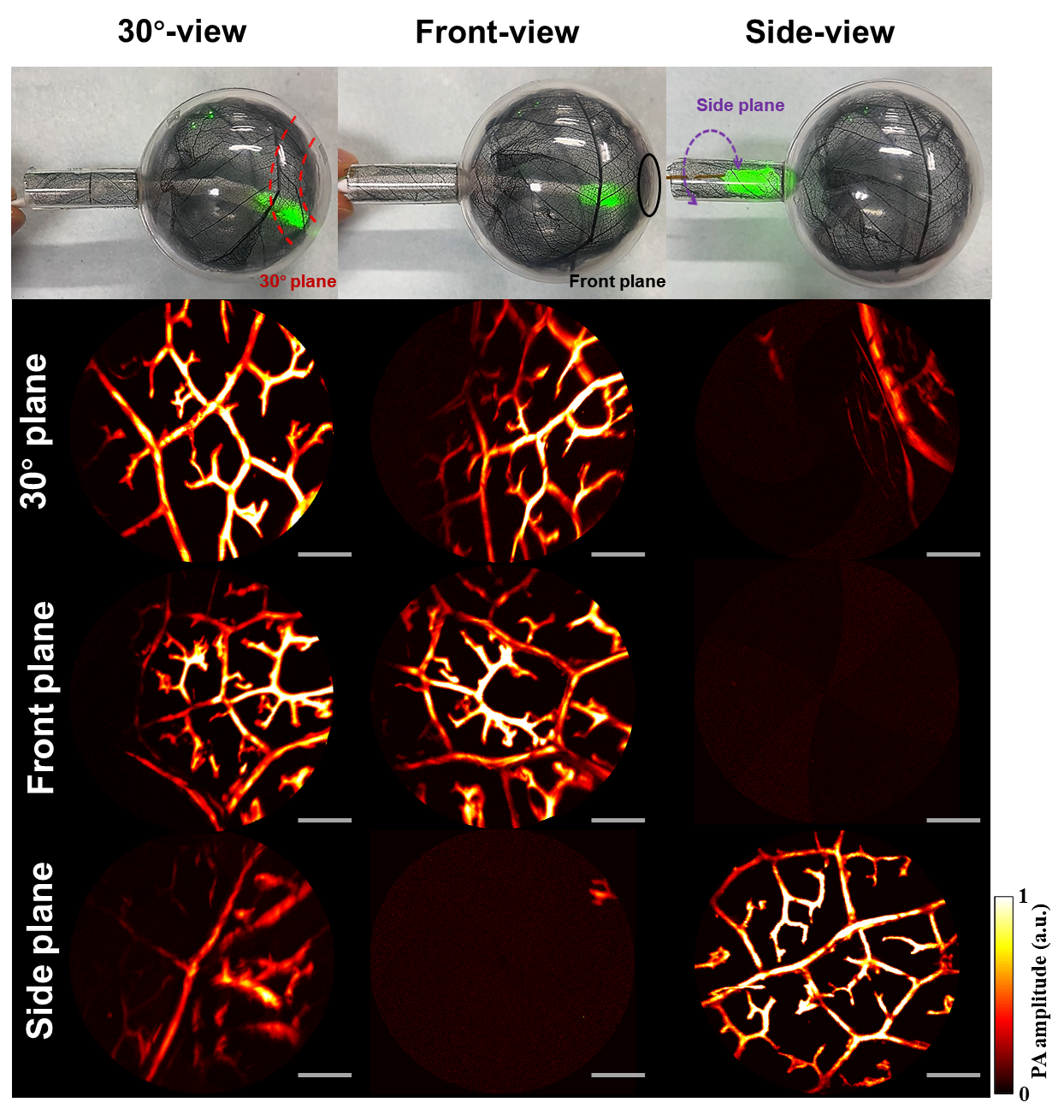
**

**Figure S3. Evaluation of imaging capabilities of three interfaces.** The 30°-view interface can be adjusted to capture images of leaf veins in the 30° plane (red dashed ring) and the frontal plane (black circle), and certain features in the lateral plane (purple arrow). The front-view interface captures images of leaf veins in the frontal and 30° planes but cannot image features in the lateral plane. The side-view interface captures images of leaf veins in the lateral plane but cannot image features in the frontal or 30° planes. Each interface demonstrates its suitability for specific imaging scenarios. Scale bars, 0.5 mm

**
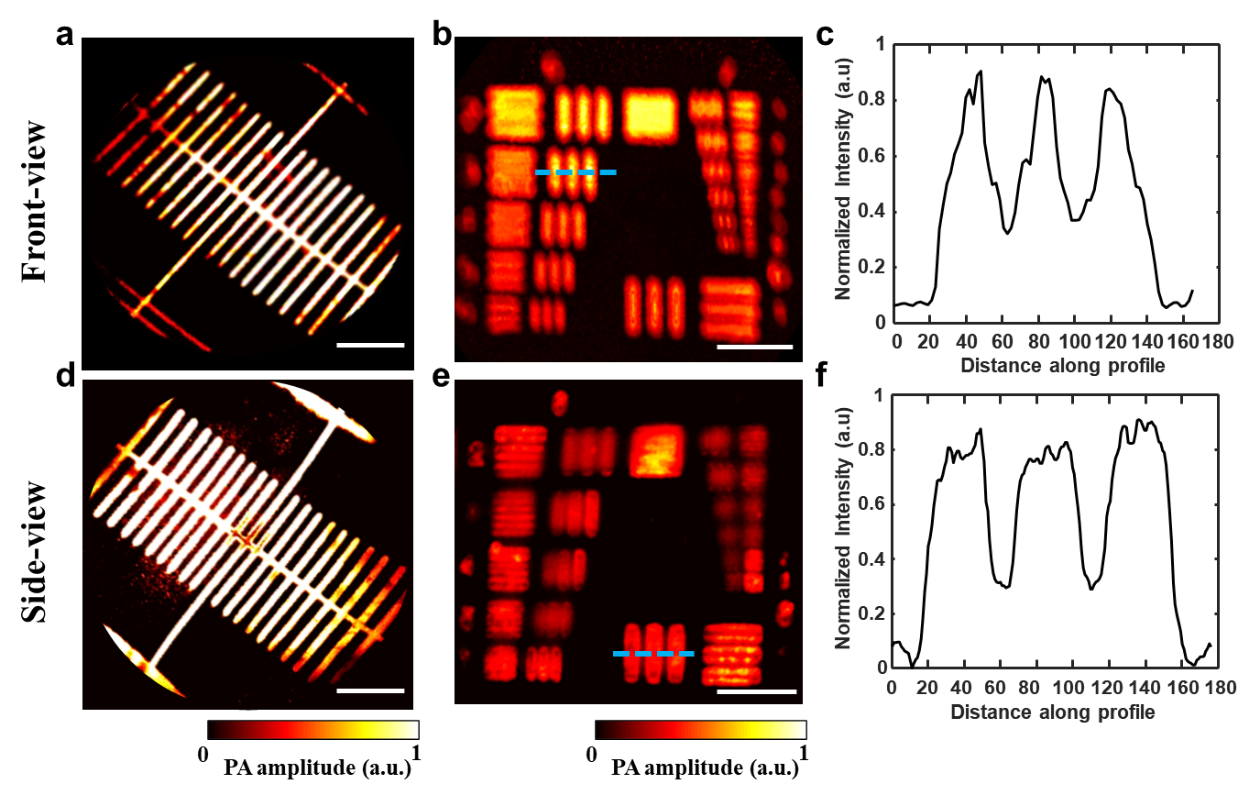
**

**Figure S4. Evaluation of FOV and resolution in side-view and front-view probes. a** Image of a reticle capytured by the front-view probe. Scale bar, 0.5 mm. **b** Image of a 1951 USAF target captured by the front-view probe. Scale bar, 50 μm. **c** Profile along the blue dashed line. The resolution is about 12.4 μm. **d** Image of a reticle capytured by the side-view probe. Scale bar, 0.5 mm. **e** Image of a 1951 USAF target captured by the side-view probe. Scale bar, 50 μm. **f** Profile along the blue dashed line. The resolution is about 15.6 μm.

**
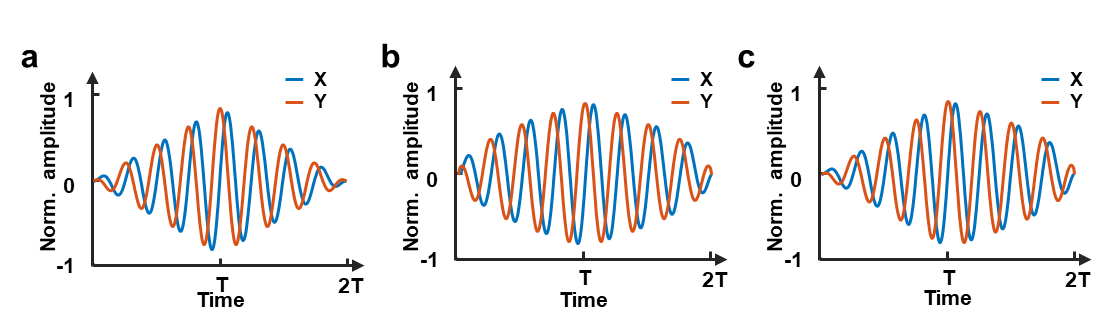
**

**Figure S5. a** The driving waveform for double spiral scanning in AS mode. **b** The driving waveform for double spiral scanning in FS mode. **c** The driving waveform for double spiral scanning in A-FS mode.

**
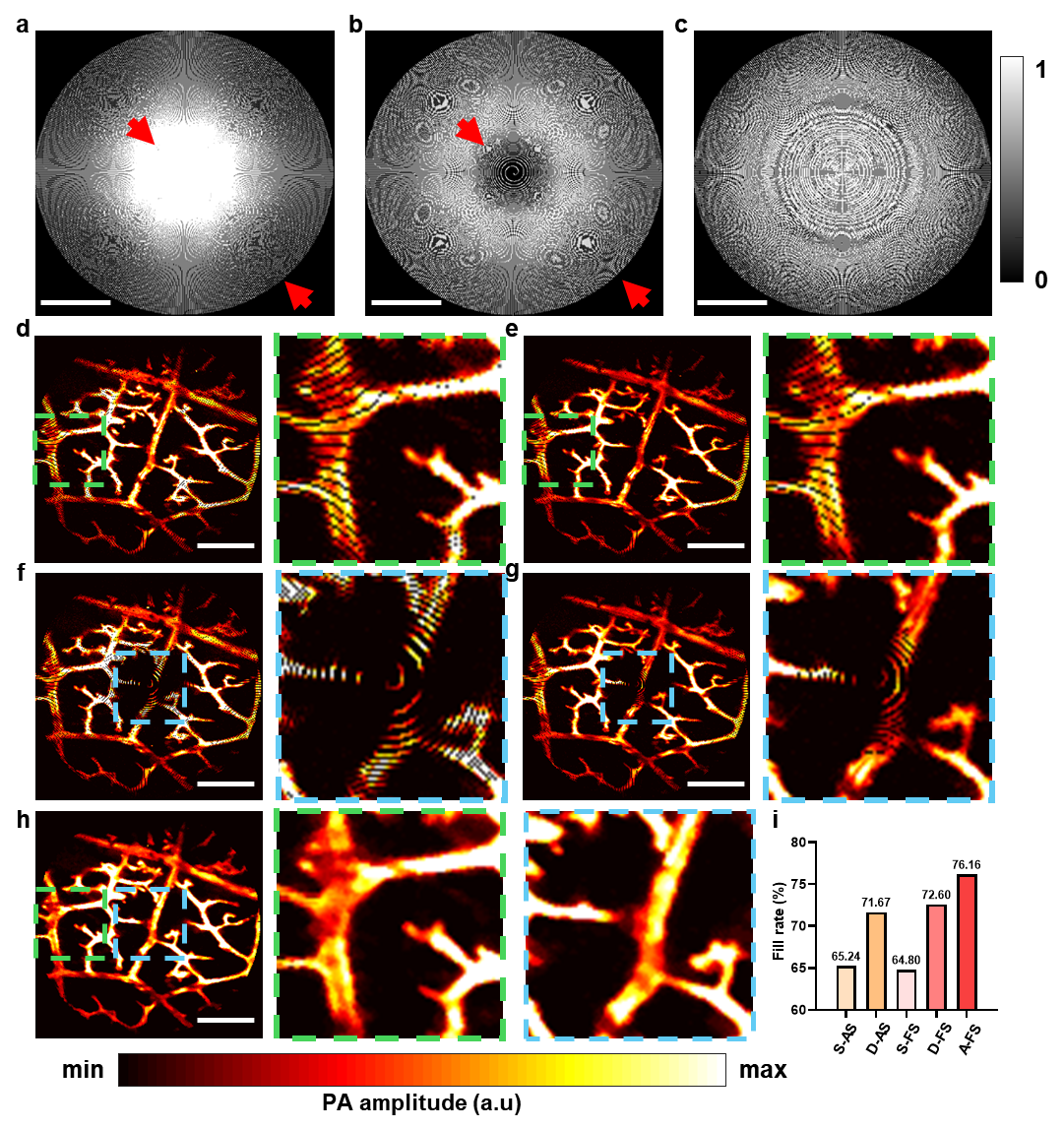
**

**Figure S6. Comparison of AS, FS and A-FS scanning mechanisms**. **a-c** The content-independent zero-point masks for AS, FS and A-FS, respectively. The effective pixels are labeled with a value of 1, and all remaining pixels are set to 0. AS scanning provides a densely sampled central region and a sparsely sampled marginal region (**a**). FS scanning provides a sparsely sampled central region, and increases the sampling rate in the marginal region (**b**). A-FS scanning achieves a more uniform sampling across the entire region (**c**). **d** Leaf vein image acquired by the single AS scan and the enlarged view in the green dashed box. **e** Leaf vein image acquired by the double AS scan and the enlarged view in the green dashed box. The double AS scan slightly alleviates undersampling at the edges. **f** Leaf vein image acquired by the single FS scan and the enlarged view in the blue dashed box. **g** Leaf vein image acquired by the double FS scan and the enlarged view in the blue dashed box. The double FS scan alleviates undersampling at the center but still suffers from insufficient sampling. **h** A-FS scan provides uniform sampling in both the center and edge. Scale bars, 0.5 mm. **i** Fill rate comparison. S-AS, single Archimedes spiral; D-AS, double Archimedes spiral; S-FS, single Fermat spiral; D-FS, double Fermat spiral. A-FS, Archimedes-Fermat spiral. A-FS improves sampling uniformity and increases the number of effective sampling points, leading to an enhanced image quality and a higher pixel filling rate.


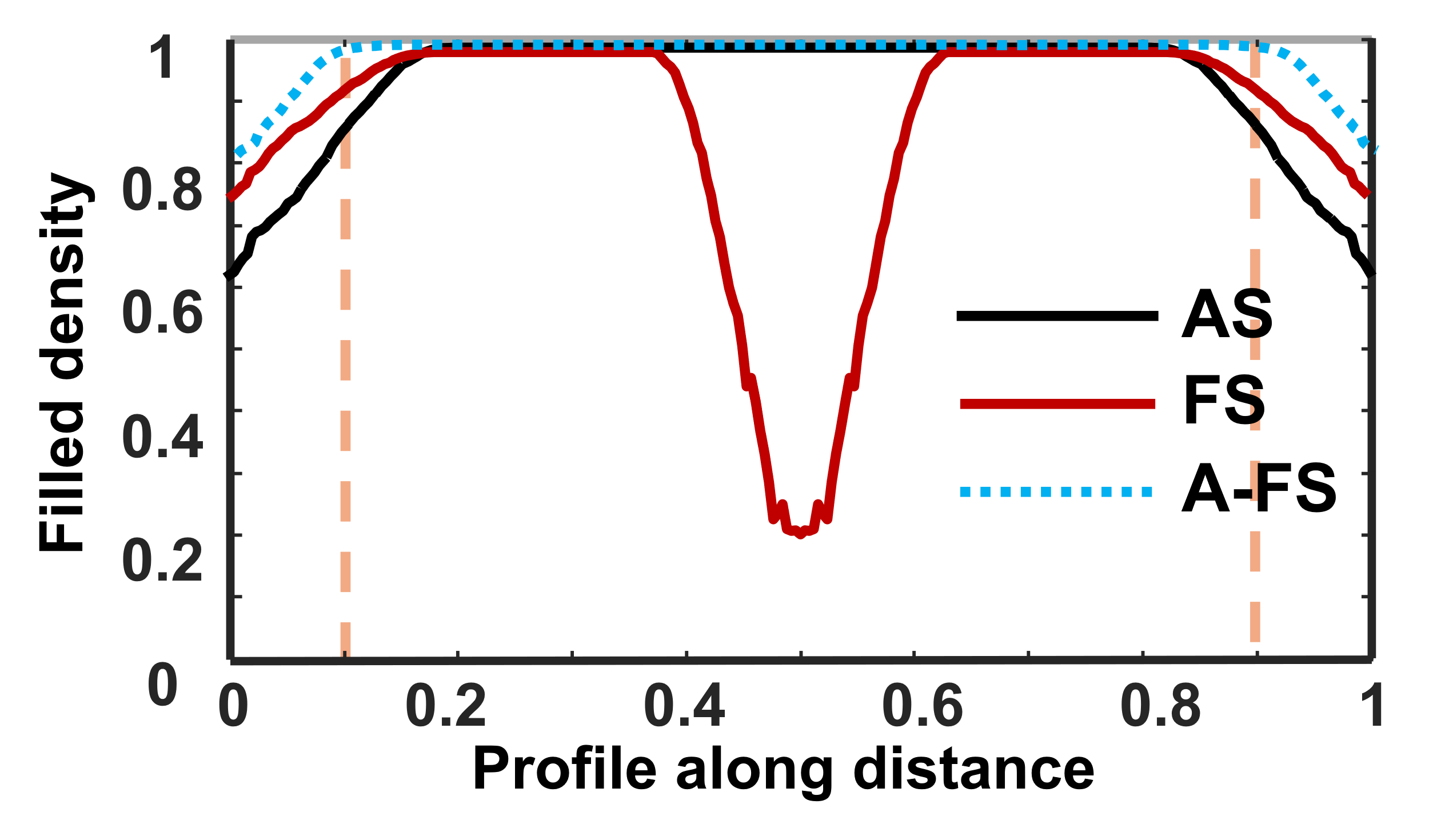


**Figure S7.** Filled density (F(r)) denotes the average sampling rate of the image along the diameter. F(r)=(S(r))/(N(r)), where S(r) denotes the number of points sampled by laser scanning on a circle with a radius r and N(r) denotes the number of points on a circle with radius r. A-FS scan expands the fully filled area in the image and enhances the filling density at the edge of the image.

**
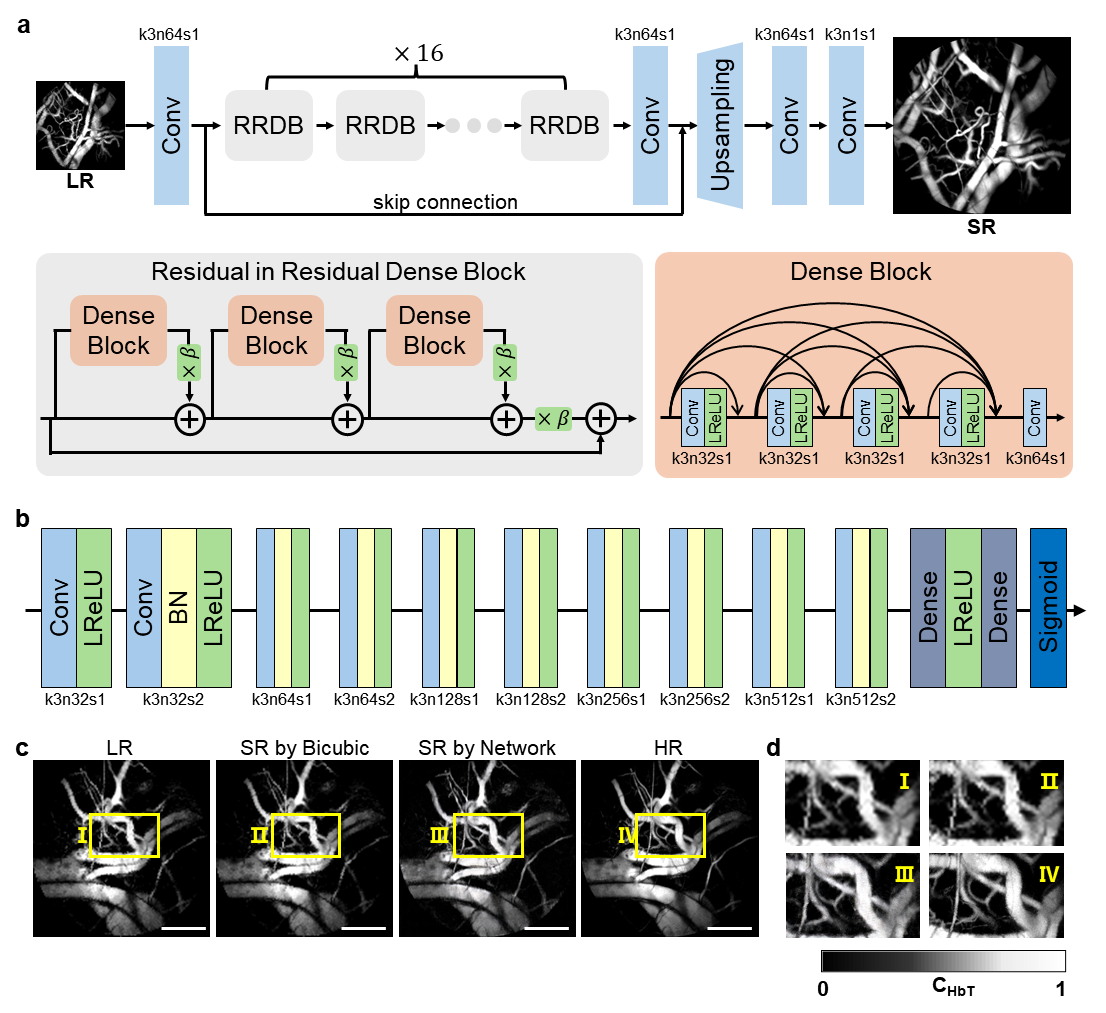
**

**Figure S8. Deep learning algorithm and performance evaluation. a,b** Architectures of Generator **(a)** and Discriminator **(b)** Networks with a corresponding kernel size (k), number of feature maps (n) and stride (s) indicated for each convolutional layer. **c** From left to right are low-resolution (LR) image, image recovered by bicubic method, image recovered by deep learning method and high-resolution (HR) image and close-up images (**d**). Scale bar, 0.5 mm.


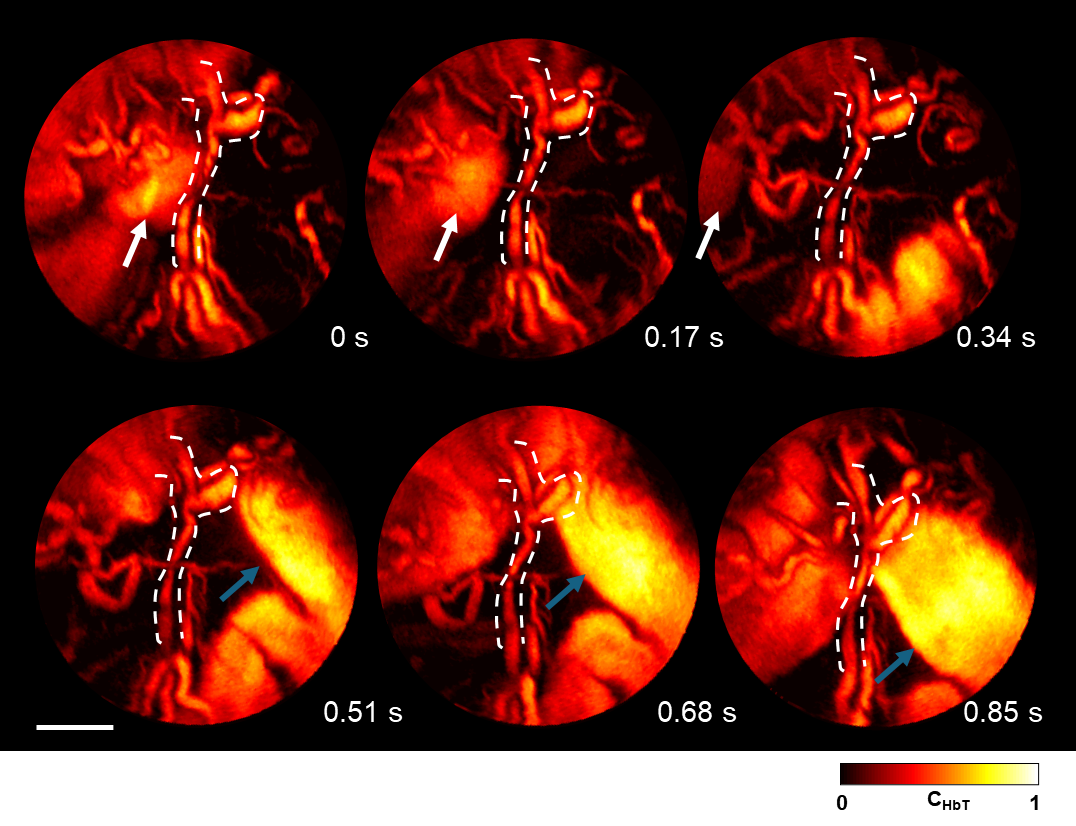


**Figure S9. Video-rate imaging of vascular dynamics in the rabbit cervix.** Single field-of-view images were captured at time intervals of 0, 0.17, 0.34, 0.51, 0.68, and 0.85 s, illustrating the motion of different vascular layers. The vascular structure outlined by the white dashed line remains steady throughout the 0.85 s duration. The vessel structure marked by the white arrow disappears gradually within the first 0.34 s, while the structure indicated by the blue arrow emerges progressively from 0.34 s. These observations demonstrate differential movement and dynamics between vascular layers within the imaging. Scale bar, 0.5 mm.


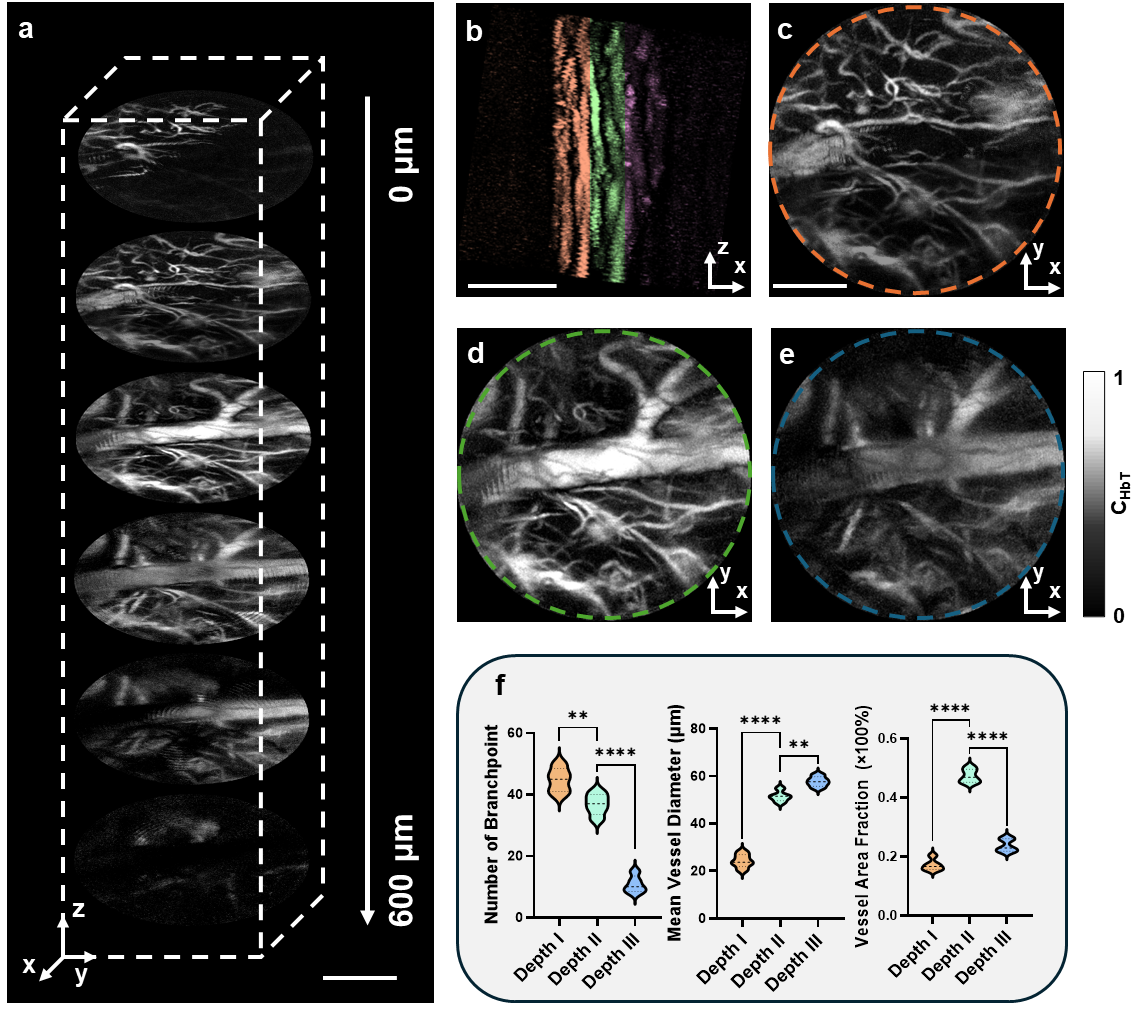


**Figure S10. 3D visualization and quantitative analysis of microvascular networks across rabbit cervix within different depths.** **a** Imaging slices of the microvasculature along the z-axis, spanning a range from 0 to 600 μm. Each image represents the maximum amplitude projection (MAP) image on the x-y plane within a depth of 100 μm. **b** MAP on the x-z plane. **c–e** Representative en face projections on the x-y plane at different depths: **c** Superficial layers (Depth I), **d** Intermediate layers (Depth II), and **e** Deep layers (Depth III). **f** Quantitatively comparisons of the number of branch points, mean vessel diameter, and vessel area fraction in Depth I, II and III. Data analysis was performed in five slices within the corresponding depth interval. **, p < 0.01; ****, p < 0.0001. Scale bars: 0.5 mm.

**
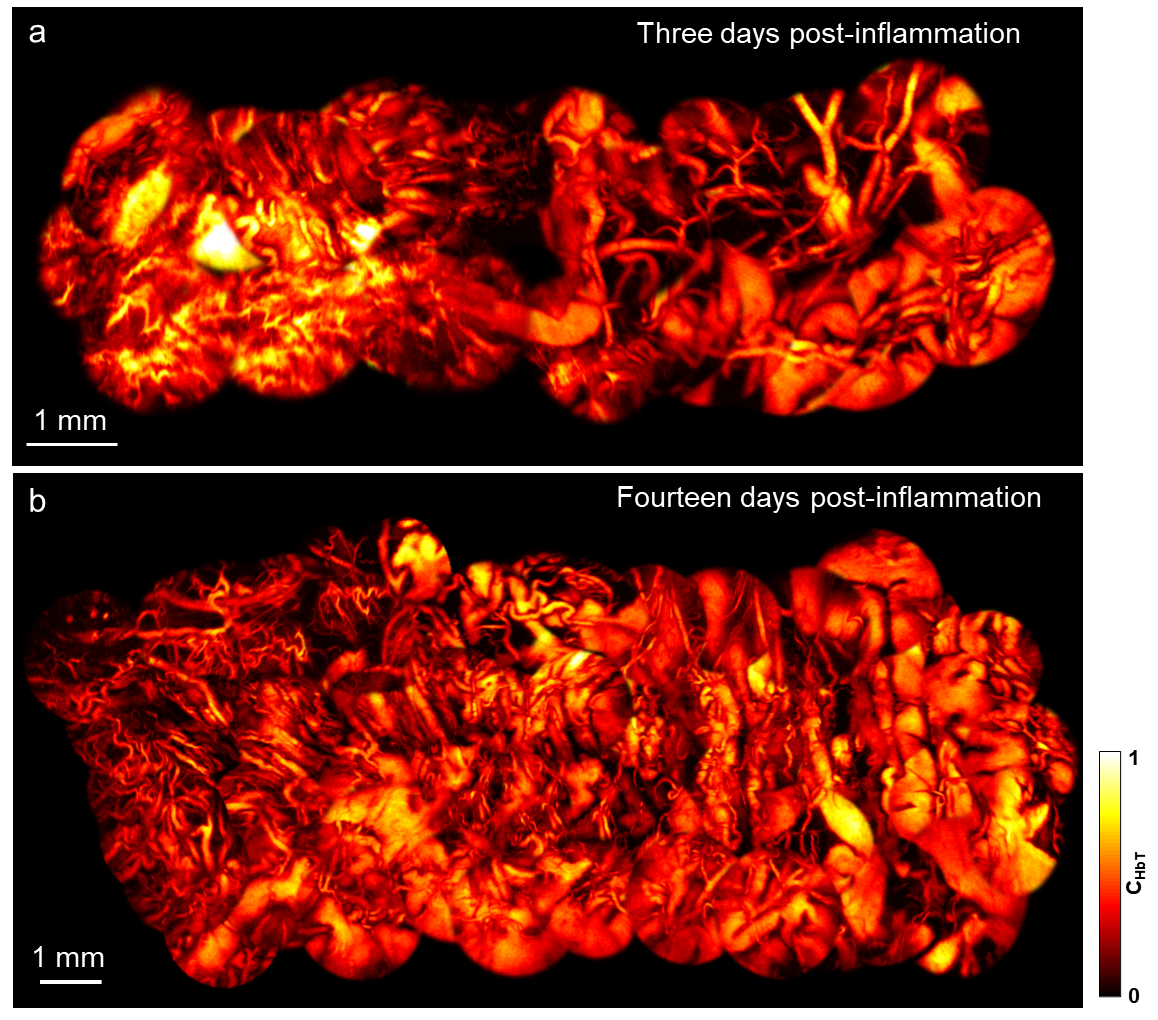
**

**Figure S11. Stitched images of a rabbit cervix microvasculature at different time points during cervicitis using Multi-PAE. a** Stitched images at three days post-inflammation illustrates angiogenesis and heterogeneity in vascular growth and morphology during the early inflammatory phase. **b** Stitched images at fourteen days post-inflammation demonstrate microvascular restoration and vessel regrowth, indicating recovery and vascular remodeling following inflammation. Scale bars: 1 mm.

**
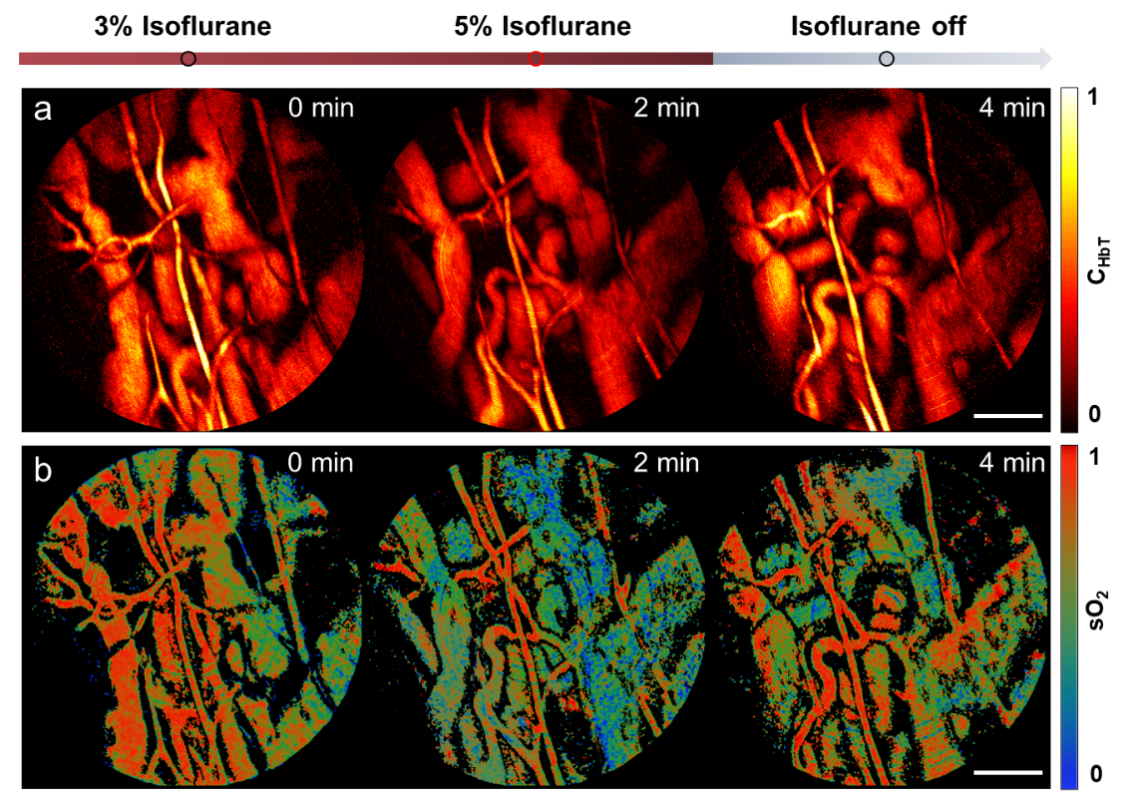
**

**Figure S12. Variations in total hemoglobin concentration (C_HbT_) and oxygen saturation (sO_2_) during hypoxia and resuscitation in the rabbit cervix.** **a** Representative images of C_HbT_ at 0, 2, and 4 minutes post the start of the experiment show vascular changes as isoflurane concentration increases from 3% to 5% and during recovery when isoflurane is turned off. **b** Corresponding sO_2_ images highlight the decrease in oxygen saturation with increased isoflurane concentration and the subsequent recovery. Scale bars: 0.5 mm.


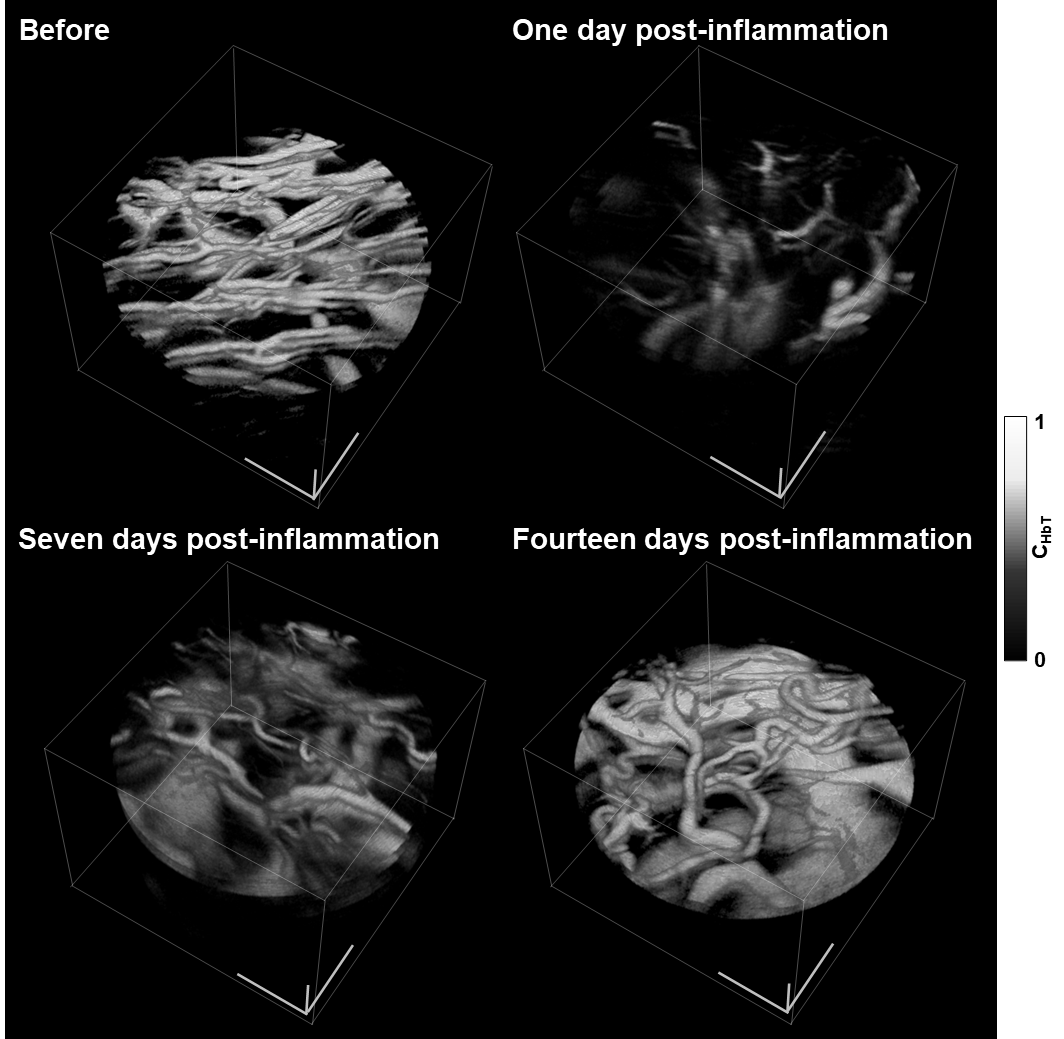


**Figure S13. 3D rendering of the vasculature in the rabbit cervix at different time points during cervicitis.** 3D images were acquired with Multi-PAE before inflammation, at one day post-inflammation, at seven days post-inflammation, and at fourteen days post-inflammation. Changes in vessel structure and density across different layers are observed, highlighting the impact of inflammation on the vascular system. Scale bars, 0.5 mm.

**
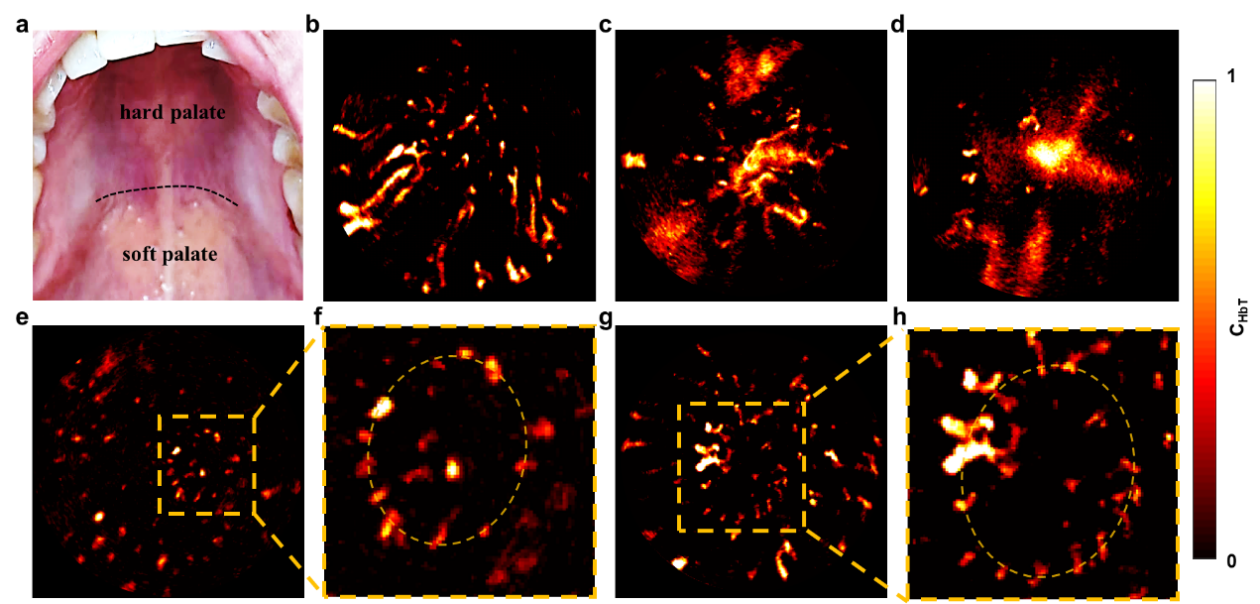
**

**Figure S14. Imaging the human hard palate. a** A photograph of the hard palate. **b-d** Various vascular structures captured in the hard palate. **e, g** Punctate blood vessels and circular blood vessel clusters in the hard palate. **f, h** Enlarged views of vessel clusters from the yellow dashed boxes.

**
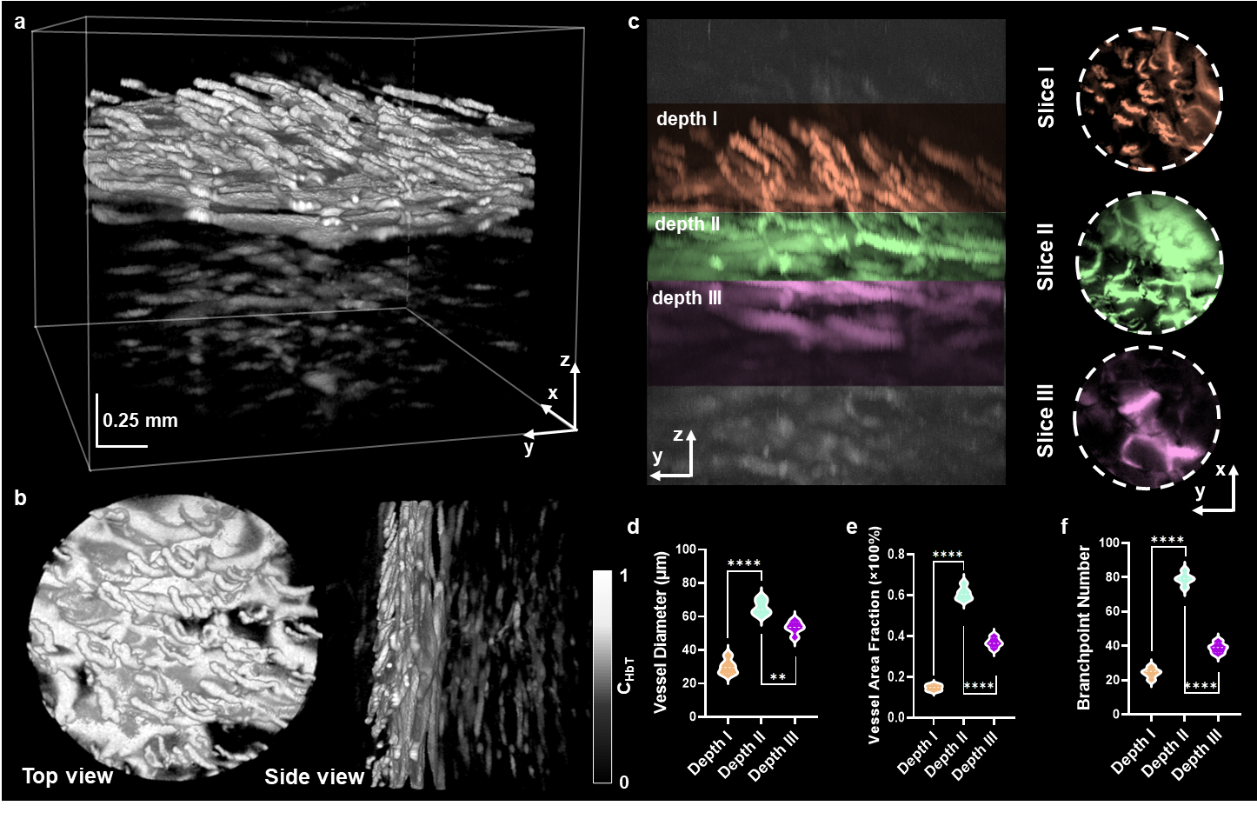
**

**Figure S15. 3D visualization of vascular structures in the cheek. a** Volumetric rendering of the vessels in the cheek. **b** Top view and side view taken from the 3D rendering. Tadpole-shaped protrusions, aligned in similar orientations, are scattered across the surface. **c** Maximum amplitude projection (MAP) of the yz cross-section from the 3D rendering (**a**), along with three representative xy cross-sectional MAPs. **d** Quantification of vascular parameters at depths Ⅰ, Ⅱ, Ⅲ. Data analysis was performed on five slices within the corresponding depth interval. Superficial blood vessels appear as small, ordered structures, while middle-layer vessels are thicker and more complex. **, p < 0.01; ****, p < 0.0001.

**
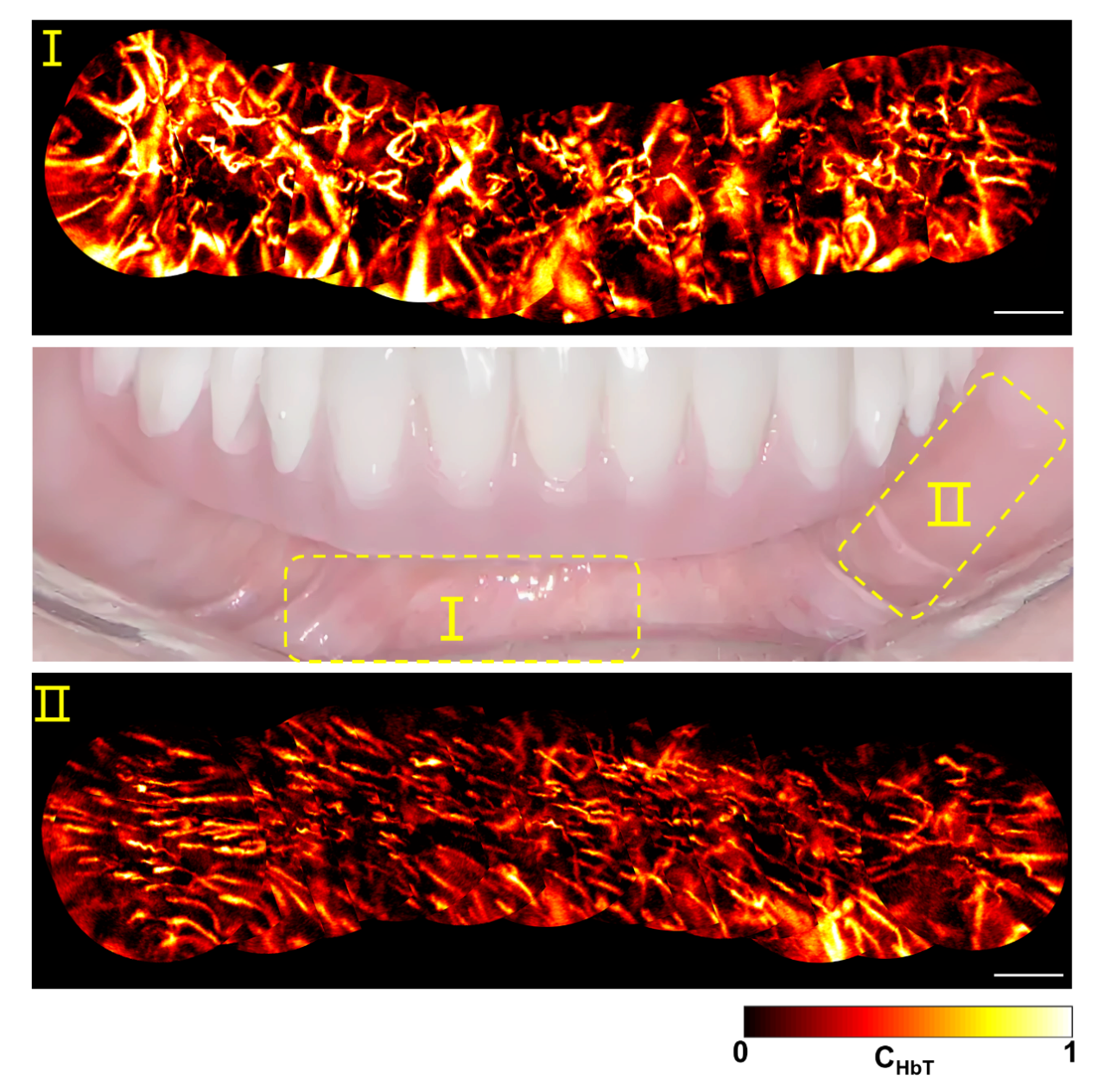
**

**Figure S16. Typical vascular structures of an oral vestibule.** The blood vessels in the frontal region (Ⅰ) primarily exhibit a vascular network pattern, while those in the lateral region (Ⅱ) predominantly exhibit a tadpole-shaped pattern.

**
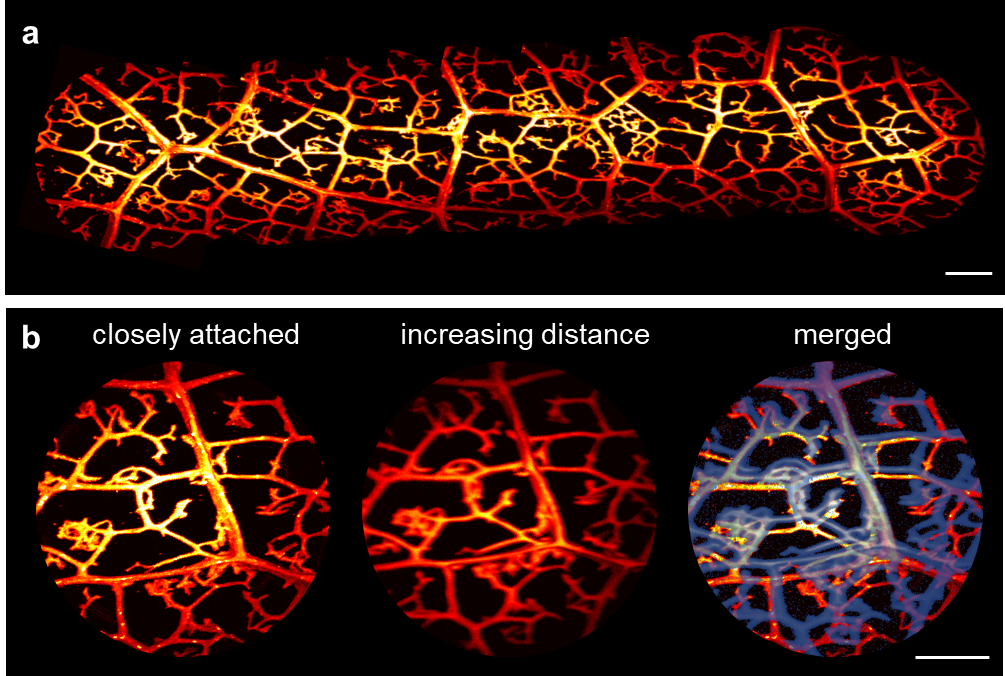
**

**Figure S17.** a Continuous acquisition and stitching of images from leaf vein phantoms. b Structural misalignment induced by variations in the distance between the imaging port and the leaf veins. Scale bars, 0.5 mm.

**Reference**

1. C. Liu, Y. Liang, L. Wang, Single-shot photoacoustic microscopy of hemoglobin concentration, oxygen saturation, and blood flow in sub-microseconds, Photoacoustics 17 (2020) 100156. <https://doi.org/10.1016/j.pacs.2019.100156>.

2. M. Li, Y. Tang, J. Yao, Photoacoustic tomography of blood oxygenation: A mini review, Photoacoustics 10 (2018) 65–73. <https://doi.org/10.1016/j.pacs.2018.05.001>.

3. R. Cao, J. Li, B. Ning, N. Sun, T. Wang, Z. Zuo, S. Hu, Functional and oxygen-metabolic photoacoustic microscopy of the awake mouse brain, NeuroImage 150 (2017) 77–87. <https://doi.org/10.1016/j.neuroimage.2017.01.049>.

4. Y. Liang, W. Fu, Q. Li, X. Chen, H. Sun, L. Wang, L. Jin, W. Huang, B.-O. Guan, Optical-resolution functional gastrointestinal photoacoustic endoscopy based on optical heterodyne detection of ultrasound, Nat Commun 13 (2022) 7604. <https://doi.org/10.1038/s41467-022-35259-5>.

5. T. Jerman, F. Pernuš, B. Likar, Ž. Špiclin, Enhancement of Vascular Structures in 3D and 2D Angiographic Images, IEEE Transactions on Medical Imaging 35 (2016) 2107–2118. <https://doi.org/10.1109/TMI.2016.2550102>.

6. T. Jerman, F. Pernuš, B. Likar, Ž. Špiclin, Blob Enhancement and Visualization for Improved Intracranial Aneurysm Detection, IEEE Transactions on Visualization and Computer Graphics 22 (2016) 1705–1717. <https://doi.org/10.1109/TVCG.2015.2446493>.

7. Y. Zhao, T. Li, H. Guo, R. Hu, L. Xi, Long-term assessment of cutaneous inflammation and treatment using optical resolution photoacoustic microscopy, Biomed. Opt. Express, BOE 14 (2023) 4775–4789. <https://doi.org/10.1364/BOE.499627>.

8. X. Wang, K. Yu, S. Wu, J. Gu, Y. Liu, C. Dong, Y. Qiao, C.C. Loy, ESRGAN: Enhanced Super-Resolution Generative Adversarial Networks, in: L. Leal-Taixé, S. Roth (Eds.), Computer Vision – ECCV 2018 Workshops, Springer International Publishing, Cham, 2019: pp. 63–79. <https://doi.org/10.1007/978-3-030-11021-5_5>.

9. K. Simonyan, A. Zisserman, Very Deep Convolutional Networks for Large-Scale Image Recognition, (2015). <https://doi.org/10.48550/arXiv.1409.1556>.

10. A. Jolicoeur-Martineau, The relativistic discriminator: a key element missing from standard GAN, (2018). <https://doi.org/10.48550/arXiv.1807.00734>.

11. D.P. Kingma, J. Ba, Adam: A Method for Stochastic Optimization, (2017). <https://doi.org/10.48550/arXiv.1412.6980>.
